# Time‐Restricted Feeding Delays Cardiovascular Aging by Increasing Acetic Acid Through Remodeling Gut Microbiota

**DOI:** 10.1111/acel.70728

**Published:** 2026-10-07

**Authors:** Man Liu, Mandi Luo, Yangyang Huang, Tianyi Ji, Yuman Wang, Qian Wang, Jie Huang, Tao Jiang, Cuntai Zhang, Tao Mi, Mian Cheng

**Affiliations:** ^1^ Key Laboratory of Vascular Ageing, Ministry of Education, Department of Geriatrics, Tongji Hospital of Tongji Medical College Huazhong University of Science and Technology Wuhan People's Republic of China; ^2^ Department of Geriatrics, Tongji Hospital of Tongji Medical College Huazhong University of Science and Technology Wuhan People's Republic of China

**Keywords:** acetic acid, gut microbiota, time‐restricted feeding, vascular aging

## Abstract

Time‐restricted feeding (TRF) restricts food intake to daily windows, yet its efficacy against vascular aging and underlying mechanisms remain unclear. Here, we demonstrate that TRF attenuates angiotension II ‐induced accelerated vascular aging by reshaping gut microbiota, stabilizing microbial diversity, and elevating the Firmicutes/Bacteroidetes ratio. TRF preserved intestinal barrier integrity, upregulated tight junction proteins (Occludin, ZO‐1, MUC2), reduced serum lipopolysaccharide, and suppressed systemic inflammation. These benefits translated into alleviated hypertension, aortic stiffness (reflected by pulse wave velocity), and pathological cardiac remodeling. Fecal microbiota transplantation recapitulated these effects, whereas antibiotic‐induced microbiota depletion abolished TRF protection; importantly, exogenous acetate supplementation fully restored the phenotypes, establishing acetic acid as the critical downstream mediator. Mechanistically, acetic acid bound to GPR43, inhibited HDAC3, increased H4K8ac acetylation, blocked NLRP3‐mediated pyroptosis, and reduced senescence markers (p53, p21, p16) in human umbilical vein endothelial cells, aorta, and heart. 16S rRNA sequencing and short ‐ chain fatty acids metabolomic profiling further identified *Limosilactobacillus* as the primary acetic acid‐producing bacterium enriched by TRF. Together, we uncover a *Limosilactobacillus*‐acetic acid‐GPR43/HDAC3/H4K8ac/NLRP3 axis, offering mechanistic insights into TRF as a non‐pharmacological strategy against age‐related cardiovascular diseases.

## Graphical abstract

Time‐restricted feeding delays vascular aging and cardiac remodeling by fostering a symbiotic gut microbiota that bolsters barrier function and amplifies short‐chain fatty acid production.

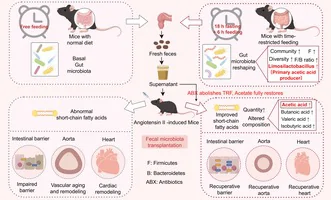

## 1 Introduction

Against the backdrop of global aging, the burden of cardiovascular disease is increasing (Joynt Maddox et al. 2024). Age‐related vascular physiological and pathological changes play a key role in the morbidity and mortality of the elderly (Lakatta and Levy 2003). Blood vessels are distributed throughout the body, and their manifestations are particularly prominent during the aging process. Vascular aging can directly or indirectly lead to the occurrence of vascular‐related diseases, such as hypertension, Alzheimer's disease, and stroke. Age is immutable, but vascular aging caused by its growth is interventionable (Ungvari et al. 2020). Exploring important targets in the process of vascular aging and carrying out targeted prevention and treatment measures to delay or even reverse vascular aging may become a feasible strategy for clinical prevention and treatment of aging and related diseases.

Diet is one of the most practical options for intervening in human aging, and time‐restricted feeding (TRF) is a new anti‐aging dietary pattern (Fontana and Partridge 2015). The classic calorie‐restricted diet slows down the rate of biological aging and improves vascular function by positively regulating cellular energy and nutrient ‐ sensing pathways and activating autophagy (Ungvari et al. 2008). This has been verified in model organisms, but has shown poor compliance in population interventions, and elderly people with normal weight are prone to decreased bone density and muscle mass (Villareal et al. 2016). The benefit of TRF is that it takes into account the anti‐aging dietary needs of special populations and avoids malnutrition problems in the elderly who do not need to lose weight (Martens et al. 2020). At the same time, current studies have also shown that TRF has many health benefits, including but not limited to the active regulation of multiple organ tissues such as liver, muscle, white adipose tissue, and brown adipose tissue, and even has the effect of extending life in fruit flies (Cienfuegos et al. 2020; Deota et al. 2023; Sutton et al. 2018; Ulgherait et al. 2021). Specifically in terms of cardiovascular disease, TRF has been shown to delay the functional decline of the fruit fly heart with age, and can also reduce the weight, blood pressure, and atherogenic lipid levels in patients with metabolic syndrome (Gill et al. 2015; Wilkinson et al. 2020). At present, there is an extreme lack of population research and basic research evidence on TRF to delay or reverse vascular aging.

Current studies have verified that the intestinal microbiota has become an important regulator of human physiology, and harmful changes in its composition and function (commonly known as dysbiosis) are associated with the occurrence and development of a variety of diseases (including cardiovascular disease) (López‐Otín et al. 2023). In particular, the intestinal microbiota plays a direct and indirect role in blood pressure regulation and vascular inflammation and sclerosis (Luqman et al. 2024; Tang, Bäckhed, et al. 2019). The human gastrointestinal tract contains a large number of microorganisms, which have a significant impact on the host's nutrition, metabolic function, intestinal development, and the maturation of the immune system and intestinal epithelial cells. Diet is one of the most important external factors that influence the composition and activity of the gut microbiota, and the interaction between diet and gut microbes plays a key role in regulating vascular health (Armet et al. 2022; Ross et al. 2024; Tang, Li, and Hazen 2019). Time‐restricted feeding has been shown to restore key microbiota, but this has not been thoroughly investigated in animals models or people subjects with vascular aging (Feng et al. 2024).

In this study, we compared the gut microbiome and metabolic changes in mice fed a normal diet (ND) or TRF. To minimize confounding hormonal variability, we utilized male mice; we acknowledge the inherent sex bias in this design and the necessity for future validation in female cohorts. Furthermore, we established an angiotensin II (Ang II)‐induced accelerated vascular aging model—a well‐established acute, single‐agent stress paradigm—to investigate the effects of TRF on pathological vascular and cardiac remodeling, the gut microbiome, and the mucosal barrier. Finally, we used fecal microbiota transplantation (FMT) in a germ‐free (GF) mouse model to elucidate the beneficial effects of TRF‐remodeled gut microbiome on the gut barrier and short chain fatty acids (SCFAs) metabolism. Our results suggest that TRF is a promising dietary therapy for delaying vascular aging and demonstrate its regulatory effects on the gut microbiota.

## 2 Materials and Methods

### 2.1 Animals

A total of 100 eight‐week‐old male C57BL/6JGpt mice used in this study were procured from Shulaibao (Wuhan) Biotechnology Co. Ltd. and housed in the specific pathogen‐free animal facility at Wuhan Tongji Hospital's Laboratory Animal Center. Additionally, 45 eighteen‐week‐old GF male C57BL/6JGpt mice were obtained from GemPharmatech Co. Ltd. and maintained in a sterile isolation laboratory. All mice were fed according to the experimental design schedule, provided with autoclaved pure water ad libitum, and exposed to a 12‐h light–dark cycle. Corncob bedding (Shulaibao, Wuhan, China) and common feed (Shulaibao, Wuhan, China; macronutrient composition: 18%~20% protein, 4%~5% fat, 50%~55% carbohydrate, 4%~5% crude fiber, and 2.9~3.1 kcal/g metabolizable energy) were changed daily. Environmental conditions were maintained at a temperature of 20°C~26°C and a relative humidity of 40%~70%. All procedures complied with the approved protocol by the Ethics Committee for Experimental Animal Welfare at Wuhan Tongji Hospital (ethical approval number: TJH‐201901010).

### 2.2 Experimental Model and Feeding Schedule

The experimental design for the animal study is depicted in Figure 1A and comprised two main phases. Initially, after a one‐week acclimatization period, 8‐week‐old male C57BL/6JGpt mice were randomly assigned to either a ND group or a TRF group for the duration of the 15‐week experiment. To establish the TRF model in accordance with murine circadian rhythms, we subjected the TRF group to a classic 16‐h fast/8‐h feed regimen (Hu et al. 2025). This was implemented by restricting food access to a 6‐h window from 18:00 to 24:00, while water was provided ad libitum outside this feeding window. Ten weeks after establishing the TRF model, both the ND and TRF groups were further randomly subdivided into two subgroups each. An Ang II‐induced accelerated vascular aging model, a well‐established acute, single‐agent model for studying stress‐induced vascular remodeling, was established by subcutaneously implanting an osmotic minipump (Alzet, model 2004, USA) containing either normal saline (NS) or Ang II (MCE, HY‐13948, USA) at a dose of 1000 ng/kg/min for a duration of 28 days (Luo et al. 2024; Min et al. 2009). This procedure followed previously established protocols (Luo et al. 2024). Mice were allocated into four groups: the normal diet with NS treatment group (ND + Sham), the normal diet with accelerated vascular aging group (ND + Ang II), the time‐restricted feeding with NS treatment group (TRF + Sham), and the time‐restricted feeding with accelerated vascular aging group (TRF + Ang II). Daily food intake was monitored, and body weight and fasting blood glucose levels were measured weekly. In another part of the experiment, male GF C57BL/6JGpt mice were employed for FMT procedure. At the conclusion of the experimental phases, the pulse wave velocity (PWV), blood pressure and cardiovascular ultrasound parameters were quantified in the mice, and tissues, feces and blood samples were collected for subsequent experiments.

**FIGURE 1:**
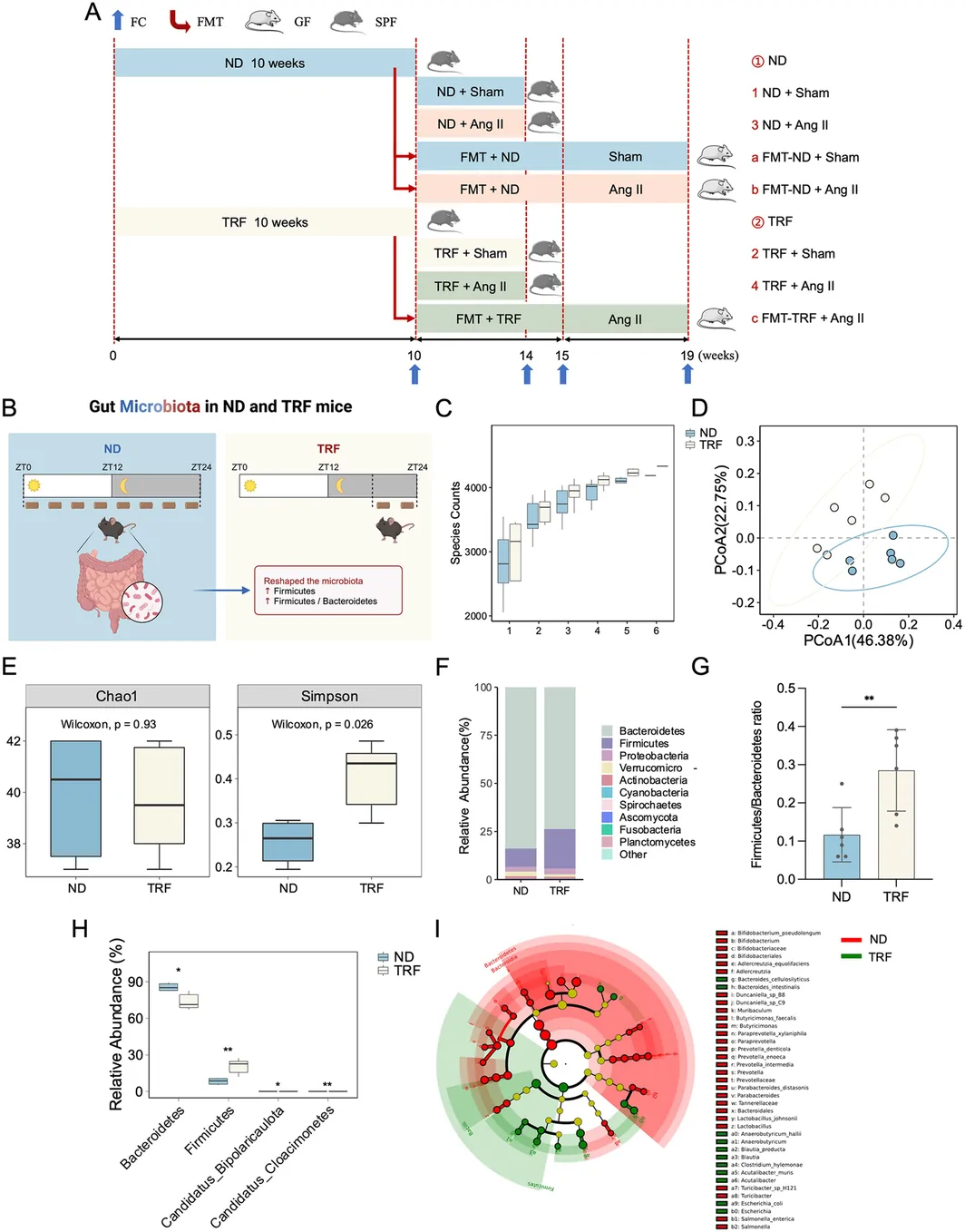
TRF contributed to beneficial gut microbiota in mice before vascular aging. (A) Schematic representation of study design. (B) Schematic diagram of the ND or TRF mice experimental design. (C) Species accumulation curve of fecal microbiota. (D) Principal Co‐ordinates Analysis (PCoA) scores based on the relative abundance of operational taxonomic units. Each symbol represents a sample. (E) α‐diversity Chao1, Simpson, and Shannon indices. (F) Dominant phyla in the ND or TRF group. (G) The column of Firmicutes/Bacteroidetes ratio (F/B ratio). (H) Differential analysis of phyla in the ND or TRF group. (I) Linear discriminant analysis effect size (LEFSe) analysis of two groups. Sample numbers of (C–I) were *n* = 6 per group. Results are expressed as mean ± SD, error bars represent SD. *p < 0.05, **p < 0.01.

### 2.3 FMT Model Construction

Mice from ND and TRF groups were used as microbiota donors for FMT experiments. And male GF C57BL/6JGpt mice were the recipient. Before FMT treatment, male GF C57BL/6 mice (18 weeks) were bred and maintained in special plastic isolators (GemPharmatech, Nanjing, China). All mice were housed under a strict 12 h light cycle (lights on at 08:00). Animals were supplied with a 50‐kGy irradiated sterile pelleted common feed and autoclaved tap water ad libitum. Bedding was replaced in all experiments every 7 days. GF mice were randomly divided into three groups. During the 15‐week experimental period, 300~350 mg of fecal pellets were collected daily from donor mice (ND and TRF group). Fecal samples were collected from the fresh mid ‐ to ‐ late portion of the interior feces to avoid contamination by urine or outer dried feces. The samples were placed in a sterile container (approximately 0.2 g per tube), then sealed, and immediately frozen at −80°C. The collected fresh feces were resuspended in 2 mL of sterile saline, homogenized vigorously, and then centrifuged (500 g, 10 min) to pellet large particles. The supernatant was immediately used for subsequent transplantation. Each group of GF mice received 200 μL of the fecal supernatant via oral gavage three times per week for 4 weeks, including a 1‐week colonization period (Goh et al. 2021; Guo et al. 2023). Subsequently, GF mice were treated with either NS or Ang II according to the previously described protocol, resulting in the following experimental groups: normal diet FMT + NS treatment group (FMT‐ND + Sham), normal diet FMT + accelerated vascular aging group (FMT‐ND + Ang II), and time‐restricted feeding FMT + accelerated vascular aging group (FMT‐TRF + Ang II). At the conclusion of the experimental phases, the PWV, BP and cardiovascular ultrasound parameters were quantified in the mice, and tissues, feces and blood samples were collected for subsequent experiments.

### 2.4 Fasting Blood Glucose and Body Weight Measurement and Oral Glucose Tolerance Test (OGTT)

For fasting blood glucose measurement, mice were fasted starting at 22:00 on the preceding day, and blood samples were collected from the tail vein at 8:00 the following morning. For the OGTT, following 10 h of fasting, mice were administered 2 g/kg glucose (Servicebio, GC205003, China) intraperitoneally. Blood samples were obtained from the tail vein at 0, 15, 30, 45, 60, 90, and 120 min after glucose injection. All blood glucose levels were further detected by a Bayer blood glucose meter (Contour TS, Germany) and Bayer blood glucose dipstick (ContourTM TS, Germany).

### 2.5 Body Weight, Body Length and Lee Index of Mice

Mice body weight was measured by an electronic scale (ZOGGI, Chaozhou, China). Then, mice were carefully positioned on the table, and its posture was adjusted to an extended position. The body length of them was measured from the tip of the nose to the anus using a standardized ruler. The Lee index was calculated according to the following formula: $\mathrm{Lee}\;\text{index}=\sqrt[{3}]{\text{body weight}\;\left(g\right)}$×10/body length (cm).

### 2.6 Mice PWV Measurement

After induction and maintenance of anesthesia with 4% and 1.5% isoflurane (Yuyan, shanghai, China) by ABS gas anesthesia machine (Yuyan, Sanghai, China), mice were placed supine on the heated electrocardiogram (ECG) conduction plate (INDUS, USA) with limbs secured to the electrodes using conductive gel (Weaver and Company, Ten 20, USA). And the ECG was ensured to be free from clutter interference. The PWV test was conducted utilizing the Small Animal Ultrasonic Doppler Flowmeter System from INDUS Corporation, USA. Ultrasound coupler gel (Cofoe, Changsha, China) was applied to the left upper chest and lower abdomen of mice near the midline of the abdominal aorta. A 20 MHz probe was utilized to detect the pulse wave of aortic arch and abdominal aorta to ascertain the optimal detection point. The real‐time pulse wave spectrogram of the optimal detection point was captured, saved, and the vertical distance between the two points was recorded. PWV was calculated according to the calculation formula: PWV = Distance between two detection points (mm)/Pulse wave conduction time between two detection points (ms) (Luo et al. 2024).

### 2.7 Mice Noninvasive Blood Pressure Measurement

Blood pressure measurement was conducted in a quiet and warm room. The CODA Multichannel Noninvasive Blood Pressure System was purchased from Kent Company, USA. Mice were fixed with a fixator in a comfortable position. The entire tail of the mouse was exposed. After placing the fixator on the heating plate, the occlusion sleeve was secured to the tail and the pressure transducer was positioned 0.5 cm from the occlusion sleeve. Then, the measurement option was activated. The machine inflated automatically until the tail blood flow was completely blocked. Immediately after, it began to deflate. The starting point of pressure alteration detected by the machine was identified as systolic blood pressure (SBP). At the moment the vein was open, the value sensor recorded was diastolic blood pressure (DBP). The mean blood pressure (MBP) is equal to (SBP + 2DBP)/3. The mean value of SBP, DBP, and MBP was calculated from multiple measurements.

### 2.8 Mice Vascular and Cardiac Ultrasound Measurement

Mice vascular and cardiac ultrasound were evaluated using a VINNO 6 VET small animal ultrasound machine (VINNO Technology, Suzhou, China). The left chest and abdomen of the mice were depilated with hair removal cream (Veet, France). After being anesthetized with isoflurane by ABS gas anesthesia machine, mice were placed supine on the operating platform with limbs fixed. Ultrasound coupler was applied evenly to the left chest and abdomen of mice. Then the ultrasound probe was placed on the left chest of the mouse. Left ventricular systolic function parameters, including left ventricle, the left ventricular ejection fraction, and left ventricular fractional shortening, were assessed in the short‐axis view. Next, the probe was positioned on the abdomen parallel to the mouse long axis and gently pressed to probe the abdominal aorta longitudinal section. Clear screenshots were saved, and the abdominal aorta external diameter was calculated.

### 2.9 Tension Detection of Isolated Vascular Rings

The responsiveness of mouse mesenteric arteries to drugs was assessed using the DMT620M in vitro microvascular tonometry system (DMT, Denmark). After the instrument was connected, physiological salt solution (PSS) was added to the bath mixed ventilation with 95% O_2_ and 5% CO_2_ at 37°C. Then, mice were sacrificed and the mesenteric arteries were quickly and gently dissociated. Two millimeters in length of the mesenteric artery was secured to a needle holder in the bath. Next, the mesenteric artery circumference was slowly adjusted and real‐time tension was monitored. At 60 min of equilibration, it was ensured that mesenteric arterial tension was controlled at an optimal initial tension of 5 mN. Then, 60 mM KCl (Solarbio, P9921, China) was used for assessing arterial ring activity. When reaching the maximal contractile response amplitude, the arterial ring was eluted with PSS three times for 10 min each. The similar magnitude of the two maximal contractile responses indicates good activity. For the vasodilation assay, the rings were pre‐contracted with phenylephrine (PE, 1 μM) to reach a stable plateau. Once a stable contraction plateau was achieved, cumulative concentration‐response curves to acetylcholine (Ach, 10^−3^ to 10^−9^ M) (MedChemExpress, HY‐B0282, USA) and sodium nitroprusside (SNP, 10^−3^ to 10^−9^ M) (MedChemExpress, HY‐B0564, USA) were obtained. To standardize the results, the relaxation responses were calculated and expressed as the percentage of the pre‐contraction tension, where the PE‐induced pre‐contraction plateau was set as 0% relaxation, and the complete return to the baseline tension was defined as 100% relaxation.

### 2.10 Mice Tissues Harvest and Processing

In the days following the completion of the modeling, fecal samples were collected in sterile cryogenic tubes and stored immediately in liquid nitrogen. Blood samples were obtained from mice via tail vein puncture. After centrifugation at 3000 rpm for 15 min at 4°C, plasma was isolated and stored at −80°C. After mice were induced and maintained under deep anesthesia with 4% and 1.5% isoflurane (Yuyan, shanghai, China), their abdominal cavities were opened with surgical scissors to expose the thoracic cavity. Pre‐cooled NS was perfused into the circulatory system at 40 mL/min until the liver color changed from dark to light. Subsequently, the aorta, heart, liver, spleen, kidneys, large and small intestine, cecum, colon, and epididymal adipose tissue were sequentially excised and weighed. Tissue specimens were fixed in 4% paraformaldehyde (Servicebio, G1101, China) or electron microscopy fixative (Servicebio, G1102, China) as required, or stored in liquid nitrogen for subsequent processing. Photographs were taken of the anal features, colon morphology, heart, and epididymal fat during sampling.

### 2.11 Serum Lipopolysaccharide (LPS) Measurement

Mouse serum LPS levels were quantified using the LPS enzyme‐linked immunosorbent assay (ELISA) Kit (FineTest, EU3126, China) following the manufacturer's protocol. Plasma samples were thawed and standard solutions were prepared. A working solution of standard/sample‐biotin‐labeled antigen was prepared and 100 μL of them was added to designated wells, followed by incubation at 37°C for 45 min. Plates were washed three times with wash buffer, each time for 1 min. Next, 100 μL of Horseradish Peroxidase (HRP)‐streptavidin working solution was added to each well and incubated at 37°C for 30 min, followed by five washes. Subsequently, 90 μL of TMB substrate was added and incubated in darkness at 37°C for 15 min. The reaction was terminated by adding 50 μL of stop solution, and absorbance was measured immediately at 450 nm. A standard curve was constructed and sample LPS concentrations were calculated accordingly.

### 2.12 Hematoxylin–Eosin (HE) Staining

Paraffin sections of aortas, hearts, small intestines, large intestines, livers, spleens, and kidneys with 4 μm thickness were baked at 60°C and subsequently deparaffinized in xylene (Aladdin, 1330‐20‐7, China). The sections were then hydrated in a gradient of ethanol from high to low. Following a five‐minute rinse with distilled water, sections were air‐dried and stained with hematoxylin (SigmaAldrich, H9627, USA) for 10 min at room temperature. After rinsing with tap water for 2 min, sections were differentiated using 1% Hydrochloric Acid‐Alcohol solution (Servicebio, G1039, China) and counterstained with 1% ammonia alcohol (Servicebio, G1040, China) to restore blue coloration. They were again washed with distilled water, air‐dried, and stained with 0.5% eosin (Servicebio, GC307022, China) for 3 min. Next, the sections were dehydrated through an ascending ethanol series and cleared in xylene. Finally, neutral resin (Servicebio, WG10004160, China) was applied for sections sealing.

### 2.13 Verhoeff's Van Gieson (EVG) Staining

Collagen Fiber and Elastic Fiber Staining Kit (EVG‐Verhoeff Method, G1597) was purchased from Beijing Solarbio company, China. Paraffin sections of the aortas, 4 μm in thickness, were sequentially subjected to baking at 60°C, deparaffinization in xylene, and hydration using a decreasing gradient of ethanol concentrations. Subsequently, the sections were stained with freshly prepared Verhoeff's staining solution for 1~3 min. After rinsing with tap water, the sections were differentiated in a 2% ferric trichloride solution. Following this, the sections were washed again with tap water and counterstained in Van Gieson's solution for 10~15 s. Then, they were washed with absolute ethanol (HUSHI, 5332, China). Dehydration was achieved by successively placing the sections in an ascending ethanol series, followed by clearing in xylene. The sections were sealed with neutral resin in the end.

### 2.14 Masson Staining

After roasting, dewaxing, and hydration, 4‐μm‐thick aortic and cardiac paraffin sections were rinsed three times with distilled water. They were then immersed overnight in potassium dichromate solution (SigmaAldrich, 1.60333, USA). Next, the sections were rinsed with tap water and stained in freshly prepared iron hematoxylin (AIOISCO, LM8H0013D, China) for 3 min. After washing with tap water and 1% hydrochloric alcohol, they were stained in ponceau acid fuchsin (AIOISCO, SS011, China) for 5~10 min. Subsequently, the sections were rinsed again with tap water, then stained with phosphomolybdic acid (BIOSIC, RY0294, China) for 3~6 min and aniline blue (Solarbio, G3668, China) for 1~3 min. Following differentiation and dehydration using 1% glacial acetic acid (SigmaAldrich, 1005706, USA) and absolute ethanol, the sections were placed sequentially in absolute ethanol, n‐butanol (SigmaAldrich, 537993, USA), and xylene for 5 min each. Finally, after drying, the sections were sealed with neutral resin.

### 2.15 Sirius Red Staining

Four‐micron‐thick heart paraffin sections were prepared through baking, deparaffinization, hydration, and rinsing. Subsequently, they were stained with Sirius red staining solution (Solarbio, S8060, China) for 15 min, rinsed with absolute ethanol, dried at 60°C, and cleared in xylene for 5 min. Finally, the sections were sealed with neutral resin.

### 2.16 Wheat Germ Agglutinin (WGA) Staining

Cardiac paraffin sections (4 μm thick) were subjected to sequential baking, deparaffinization, hydration, and rinse. Antigen retrieval was performed using an EDTA‐based buffer (pH 8.0) (Solarbio, C1033, China) via microwave‐mediated retrieval. After natural cooling, sections were washed three times with phosphate buffer saline (PBS) on a shaker for 5 min each. Once the sections were dried, a circle was drawn around the tissue using a histochemical pen (Solarbio, YA0310, China) and the diluted iFluor 488 WGA staining solution (Solarbio, I3300, China) was applied. The sections were incubated at 37°C in the dark for 1 h. And then, after three more PBS washes, they were counterstained with 4,6‐diamino‐2‐phenyl indole (DAPI) (Solarbio, C0065, China) for 10 min in the dark at room temperature. Following another set of three PBS washes, the sections were treated with autofluorescence quencher solution B (Servicebio, G1221, China) for 5 min. At last, after rinsing with running water, sections were sealed with an anti‐fluorescence quencher (Beyotime, P0126, China). Images were acquired using fluorescence confocal microscopy (Nikon, Japan).

### 2.17 Periodic Acid‐Schiff (PAS) Staining

PAS staining was performed on paraffin sections of large and small intestine tissues using a PAS staining kit (Servicebio, G1008, China) according to the manufacturer's instructions. First, sections were baked, deparaffinized, hydrated, and washed. They were then immersed in solution B for 10~15 min staining, rinsed with water, incubated in solution A for 30 min in darkness. Subsequently, after rinsing with water again, the sections were stained in solution C for 30 s. They then underwent successive treatments including differentiation in 1% Hydrochloric Acid‐Alcohol solution, rinsing with water, returning to blue with ammonia alcohol, and a final water rinse. Dehydration was performed using three changes of absolute ethanol, followed by clearing in xylene. Finally, neutral resin was applied to sections sealing.

### 2.18 Transmission Electron Microscopy (TEM)

Fresh aortic and intestinal tissues were promptly and gently harvested and fixed in electron microscope fixative (Servicebio, G1102, China) for 2 h. The tissues were then rinsed three times with PBS for 15 min each. Electron microscopy specimens were prepared following established protocols (Heegaard et al. 2007). Tissues ultrastructural changes were examined under a transmission electron microscope (JEOL, Japan), focusing on distribution and organization of elastic and collagen fibers in vascular tissues and the morphology of goblet cells and intercellular junctions in intestinal tissues. Typical images were captured.

### 2.19 Tissue Immunofluorescence Staining

Colon paraffin sections were baked, deparaffinized, and hydrated. Sections were washed three times with PBS for 5 min each on a shaker, followed by antigen retrieval in sodium citrate buffer (pH 6.0) (Servicebio, G1202, China) using microwave treatments. After cooling, they were washed again with PBS, and a circle was drawn around the colon tissue using a histochemical pen. Next, they were incubated with 3% hydrogen peroxide for 20 min to quench endogenous peroxidase activity, followed by blocking with 3% bovine serum albumin for 30 min at room temperature. Subsequently, sections were incubated with mucin 2 (MUC2) (Abclonal, A14659, China), occludin (Proteintech, 66378‐1‐Ig, USA), and zonula occludens‐1 (ZO‐1) (Abclonal, A0659, China), primary antibodies overnight at 4°C. After washing with PBS, FITC/Cy3/−conjugated fluorescent secondary antibodies were added to the sections and incubated for 60 min at room temperature to avoid light. Finally, sections were rinsed with PBS and sealed with an anti‐fade mounting medium containing DAPI (Abbkine, BMU107, USA). Specimens were observed and photographed under a fluorescence confocal microscope.

### 2.20 Western Blot

Aortic and heart segments were ground and lysed in enhanced RIPA buffer, then centrifuged at high speed. The supernatant was collected, and protein concentration was determined using a BCA kit (Beyotime, P0012S, China). Samples were adjusted to equal concentrations with water, mixed with 5× loading buffer, and boiled for 5 min. Equal volumes of proteins were loaded onto 10% polyacrylamide gel and electrophoresed at 150 V for 1 h. The proteins were transferred to a 0.45 μm polyvinylidene fluoride membrane at 230 mA for 100 min. The membrane was blocked with 5% skim milk for 1 h at room temperature, washed three times with tris buffered saline plus 0.5% Tween‐20 (TBST) for 7 min each, and incubated overnight at 4°C with primary antibodies against p53 (Cellsignal, #9282, USA), p21 (Santa Cruz, sc‐6246, USA), NLR family pyrin domain containing 3 (NLRP3) (Proteintech, 30109‐1AP, China), gasdermin D (GSDMD) (Cellsignal, #39754，USA), caspase1 (CASP1) (HUABIO, HA722222, China), tumor necrosis factor alpha (TNFα) (Proteintech, 17590‐1‐AP, China), interleukin 6 (IL6) (Proteintech, 25404‐1‐ap, China), interleukin 1 beta (IL1β) (Abcam, ab234437, USA), hydroxycarboxylic acid receptor 2 (GPR109A) (Absin, abs149251, China), free fatty acid receptor 2 (GPR43) (Proteintech, 19952‐1AP, China), free fatty acid receptor 3 (GPR41) (Proteintech, 66811‐1‐Ig, China), histone deacetylase 3 (HDAC3) (Abclonal, A28114, China), Histone H4 Lysine 8 Acetylation‌ (Abclonal, A28114, China) and glyceraldehyde‐3‐phosphate dehydrogenase (GAPDH) (Abbkine, ABL1020, USA). After washing with TBST again, the membrane was incubated with HRP labeled sheep anti‐rabbit/mouse secondary antibody (Solarbio, SE131/SE134, China) for 1 h at room temperature. Following another TBST wash, the membrane was exposed to Enhanced Chemiluminescence developer solution (Abbkine, BMU102, USA) for 30 s and photograded.

### 2.21 Metagenomic Sequencing Analysis of Intestinal Microorganisms

Mouse fecal samples from ND + Sham, TRF + Sham, ND + AngII, TRF + AngII, FMT‐ND + Sham, FMT‐ND + Ang II and FMT‐TRF + Ang II group were collected, and microbial genomic DNA was extracted using the FastPure Stool DNA Isolation Kit (Magnetic Bead) (MJYH, T10‐100, China). The DNA concentration and purity were measured with a spectrophotometer (Thermofisher, USA). One percent agarose gel electrophoresis (Thermofisher, 16550100, USA) was used for confirming its integrity. DNA was sheared using the Covaris M220 instrument (Gene Corporation, China). And fragments approximately 350 base pairs in length were selected for paired‐end (PE) library construction. Libraries were prepared using the NEXTFLEX Rapid DNA‐Seq kit (Bioo Scientific, 5144‐08, USA), including adapter ligation, magnetic bead enrichment, PCR amplification, and purification. Metagenomic sequencing was performed on the MGISEQ 2000 platform (BGI Shenzhen, China) with paired‐end 150 bp reads (PE150). During the bioinformatics analysis, we used fastp (version 0.23.4), BWA (version 0.7.17), MEGAHIT (version 1.2.9), CD‐HIT (version 4.8.1), and SOAPnuke (version 1.2) for quality control by filtering raw data. Subsequently, host‐derived sequences were identified and removed by comparing with reference sequences, generating clean data. Functional and species annotations of gene sequences were performed using Diamond (version 4.6.8). Based on gene, species, and functional abundance tables, we conducted a comprehensive analysis of fecal tissue samples using the BGI online platform (BGI Shenzhen, China). This included visualizing the distribution of genes, species, and functions, calculating both Alpha and within‐group Beta diversity indices, performing abundance clustering and sample clustering analyses, and identifying significant differences in species and functional compositions among the fecal samples.

### 2.22 Targeted Metabolomics Analysis of Gut Microbes

Mouse feces from FMT‐ND + Sham, FMT‐ND + Ang II, FMT‐TRF + Ang II, Ang II, TRF+ Ang II， TRF + Ang II+ Antibiotics‌ (ABX), TRF + Ang II + ABX + Acetate group were collected, and a 20 mg sample was placed in a 2 mL grinding tube. Then, 800 μL of 0.5% phosphoric acid solution (Macklin, P816342, USA) was added. Samples were cryogenically ground for 3 min at 50 Hz and ultrasonicated for 10 min. After centrifugation at 13,000 g for 15 min at 4°C, 200 μL of the supernatant was transferred to a 1.5 mL tube and mixed with an equal volume of n‐butanol (Macklin, B803398, USA) for extraction. Following a 10‐s vortex and low‐temperature ultrasonication for 10 min, samples were centrifuged again at 13,000 g for 5 min at 4°C and the supernatant was prepared for analysis. Subsequently, a mixture of eight SCFAs standards (acetic acid, propanoic acid, butanoic acid, isobutyric acid, valeric acid, isovaleric acid, hexanoic acid, and isohexanoic acid) was diluted with n‐butanol (HPLC grade) to prepare mixed standard solutions at varying concentrations. Similarly, 2‐ethylbutyric acid (internal standard) was diluted with n‐butanol to prepare internal standard solutions at different concentrations. Sample loading and analysis were conducted using a GC–MS analytical instrument (Agilent, 8890B‐5977B GC/MSD, USA). The default parameters of the MassHunter Quantitative Analysis software (version 10.0.707.0) were utilized to automatically identify and quantify the ion fragments of the target SCFAs. The detected concentration of each sample was calculated based on the standard curve, and the actual content of SCFA (μg/mL) in the samples was determined (Hsu et al. 2019). The data were analyzed through the free online platform of majorbio cloud platform (cloud.majorbio.com) (MJYH, Shanghai, China).

### 2.23 Mice Blood Biochemical Indexes Detection

Mice plasma samples were collected as above. Serological biochemical indicators were automatically determined by an automatic biochemical instrument (Chemray800, Shenzhen Leidu Life Technology, China). After preparing the reagents, the corresponding parameters were set on the automatic biochemical instrument, and the results were exported. Some serum indicators such as insulin were detected by an ELISA kit (Wuhan Bio‐swamp, MU30432, China), and the detection method was according to the instructions of the kit. Finally, the standard curve was made according to the concentration and OD value of the standard substance, and then the standard curve equation was used to calculate the index concentration in the serum sample.

### 2.24 Statistical Analysis

Semi‐quantitative analysis of image grayscale values and fluorescence intensities were performed using Image‐Pro Plus (version 6.0, USA). Data statistical analysis was conducted with Statistical Product and Service Solutions (SPSS, version 22.0, USA). Statistical analysis plots were generated using GraphPad Prism (version 10.0, USA) and R language (version 4.4.3). Normality was assessed using the Shapiro–Wilk test, and normally distributed continuous variables are expressed as mean ± standard deviation (SD). For multiple group comparisons, Analysis of variance followed by Tukey's Honestly Significant Difference test was employed. For comparisons between two groups, an independent samples *t*‐test was utilized. Significance levels are denoted as follows: *p < 0.05, **p < 0.01, ***p < 0.001, ****p < 0.0001, indicating statistically significant differences; ns indicates *p* ≥ 0.05, representing nonsignificant differences.

## 3 Results

### 3.1 TRF Shapes More Beneficial Intestinal Microbiota in Mice Before Accelerated Vascular Aging Induced by Ang II

Prior to accelerated vascular aging model construction, this study investigated the effects of TRF and ND on the fecal microbiota of mice following a 10‐week dietary intervention (Figure 1A,B), as well as TRF's impact on basic metabolic parameters such as body weight and fasting blood glucose. Although TRF reduced the feeding window, it increased short‐term food intake without significant differences in caloric intake between the two groups (Figure S3A). TRF showed negligible effects on body weight, body mass index (Lee index), fasting blood glucose, or epididymal fat mass (Figure S3B–E). Given TRF's reported efficacy in improving insulin sensitivity in humans with metabolic syndrome, an OGTT was additionally performed. However, no differences in glucose tolerance were observed between TRF and ND groups, likely due to the absence of disease‐related metabolic stress (Figure S3F,G). As shown in Figure 1C,D, fresh fecal samples from both groups underwent metagenomic sequencing. Unweighted PCoA revealed distinct clustering between ND and TRF groups. The Simpson index was significantly higher in the TRF group, while the Chao1 index showed no significant difference, suggesting that TRF enhanced microbial community evenness and diversity (Figure 1E). At the phylum level, both groups exhibited Bacteroidetes and Firmicutes dominance, with TRF mice displaying higher Firmicutes abundance and an elevated Firmicutes/Bacteroidetes ratio (Figure 1F–H). Previous studies indicate that a higher Firmicutes/Bacteroidetes ratio in human microbiota correlates with age‐related stability (Du et al. 2021). LEfSe further identified differential taxa dominance between groups at phylum and genus levels (Figure 1I).

### 3.2 TRF Reduces Blood Pressure and Ameliorates Vascular Stiffness in Ang II‐Induced Accelerated Vascular Aging Mice

Given the critical influence of feeding on glucose metabolism and body weight, physiological parameters were monitored throughout the accelerated vascular aging modeling period. Body weight remained comparable across groups, with TRF showing no weight‐reducing effects (Figure S3H,J). Fasting blood glucose, glucose tolerance (Figure S3I,K; Figure 2E), insulin secretion, serum insulin, and glycated serum protein levels all fell within normal ranges and showed no significant intergroup differences (Table S1). Additionally, serological markers of liver function (alanine aminotransferase, aspartate aminotransferase) and kidney function (blood urea nitrogen) remained unchanged. No abnormalities were observed in the gross or histopathological morphology of the liver, kidneys, or spleen (Figure S3L–N). Key hallmarks of accelerated vascular aging include vascular wall thickening, reduced elasticity, and clinical manifestations such as increased PWV, elevated SBP, and widened pulse pressure. Noninvasive blood pressure measurements revealed that the ND + Ang II group exhibited significantly higher SBP, DBP, and MBP compared to controls. Notably, TRF intervention had no effect on blood pressure in ND‐fed mice but significantly attenuated Ang II‐induced hypertension (Figure 2A–C). PWV, the gold‐standard metric for vascular stiffness (Marshall et al. 2024), was elevated in the ND + Ang II group versus ND + Sham controls. TRF intervention markedly reduced aortic PWV in Ang II‐treated mice (Figure 2H; Figure S3O). Vascular weight, another indicator of aging severity, aligned with PWV trends, further supporting TRF's ability to mitigate age‐related vascular stiffening (Figure 2D). Ultrasound imaging of abdominal aortic diameter showed enlargement in ND + Ang II mice, which was attenuated by TRF (Figure 2F,G). Histopathological staining (H&E, EVG, Masson) of aortic sections revealed luminal expansion, elastic fiber fragmentation, and collagen deposition in the ND + Ang II group, consistent with ultrasound findings (Figure 2J–L,N). Increased intima‐media thickness (IMT), a structural marker of accelerated vascular aging, corroborated these pathological changes (Figure 2I). TEM demonstrated disorganized collagen fibers, degraded elastic fibers, and collagen infiltration in the extracellular matrix (ECM) of ND + Ang II mice, all of which were ameliorated by TRF (Figure 2M). At the molecular level, Western blot analysis confirmed elevated expression of senescence markers p21, p16, and p53 in the ND + Ang II group. TRF intervention suppressed this senescence‐associated protein upregulation (Figure 2O). Collectively, these pathological and molecular findings demonstrate that TRF alleviates Ang II‐induced hypertension, reduces vascular stiffness, and delays accelerated vascular aging, likely through attenuation of pathological vascular remodeling.

**FIGURE 2:**
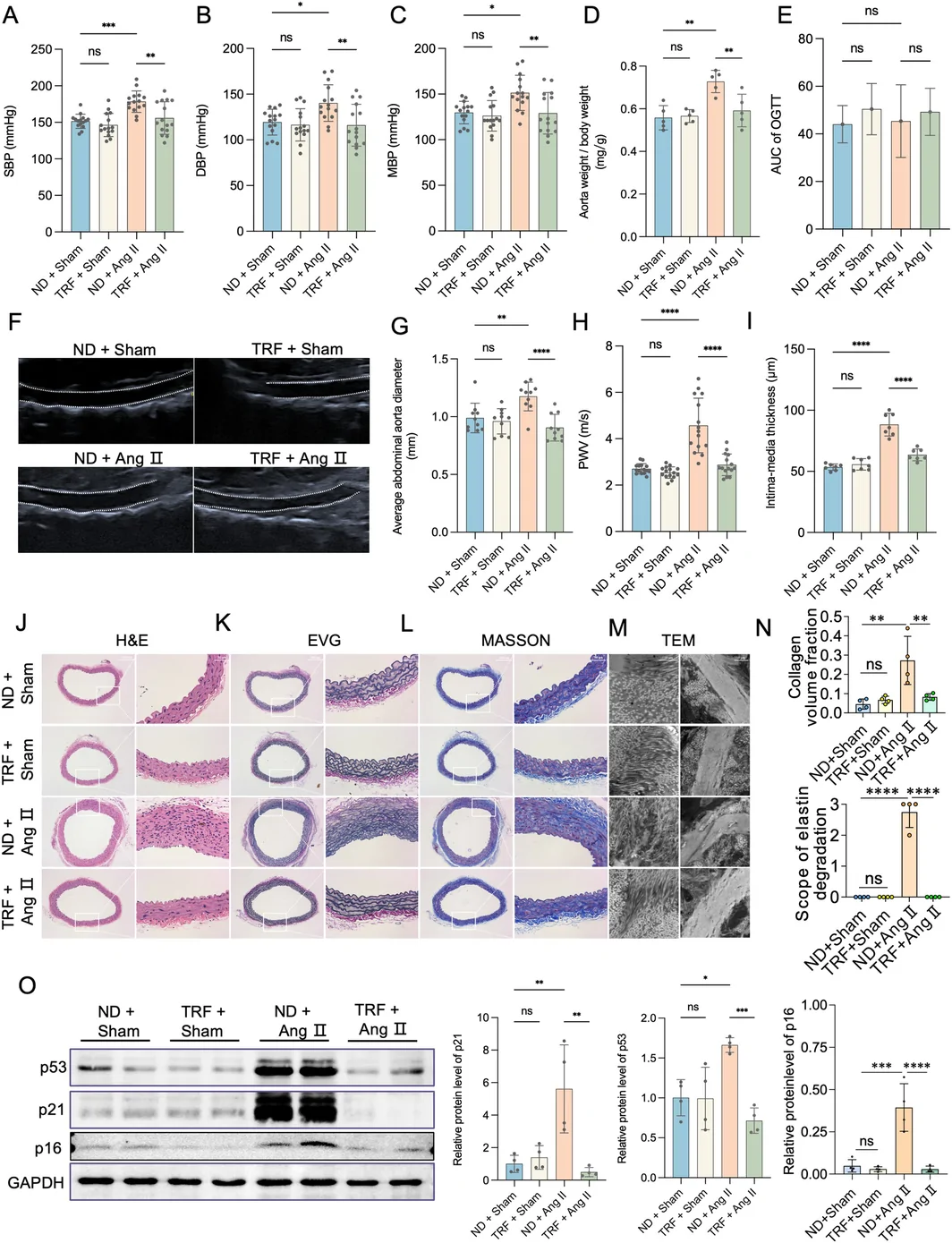
TRF alleviated angiotensin II‐induced vascular remodeling. (A) SBP of Ang II‐induced mice fed with ND or TRF. (B) DBP of Ang II‐induced mice fed with ND or TRF. (C) MBP of Ang II‐induced mice fed with ND or TRF. (D) The aorta‐weight/body‐weight ratio of Ang II‐induced mice fed with ND or TRF, *n* = 5 per group. (E) Area under curve (AUC) of OGTT (mean ± SD). (F) Representative images of abdominal aorta ultrasound. (G) Diameter of abdominal aorta of Ang II‐induced mice fed with ND or TRF, *n* = 10 per group. (H) PWV of Ang II‐induced mice fed with ND or TRF. (I) Quantification of intima‐media thickness, *n* = 7 per group. (J–L) Representative images of the abdominal aorta stained with HE, EVG and Masson, *n* = 4 per group. (M) Observation of collagen and elastic fiber arrangement in vascular wall by TEM, scale bar = 10 μm, *n* = 4 per group. Left, the cross section of collagen fibers. Right, the longitudinal section of elastic fibers. (N) Statistical analysis diagram of collagen deposition (Masson) and elastic fiber damage (EVG). (O) Western blot plots and statistical analysis of p53, p16 and p21 protein levels, *n* = 4 per group. Sample numbers were *n* = 15 per group for (A–C), (E) and (H). Results are expressed as mean ± SD, error bars represent SD. The scale bars of HE, EVG, and Masson staining photographs were 200 μm (low power field) or 50 μm (magnification field). *p < 0.05, **p < 0.01, ***p < 0.001, ****p < 0.0001. ns, not significant.

**FIGURE 3:**
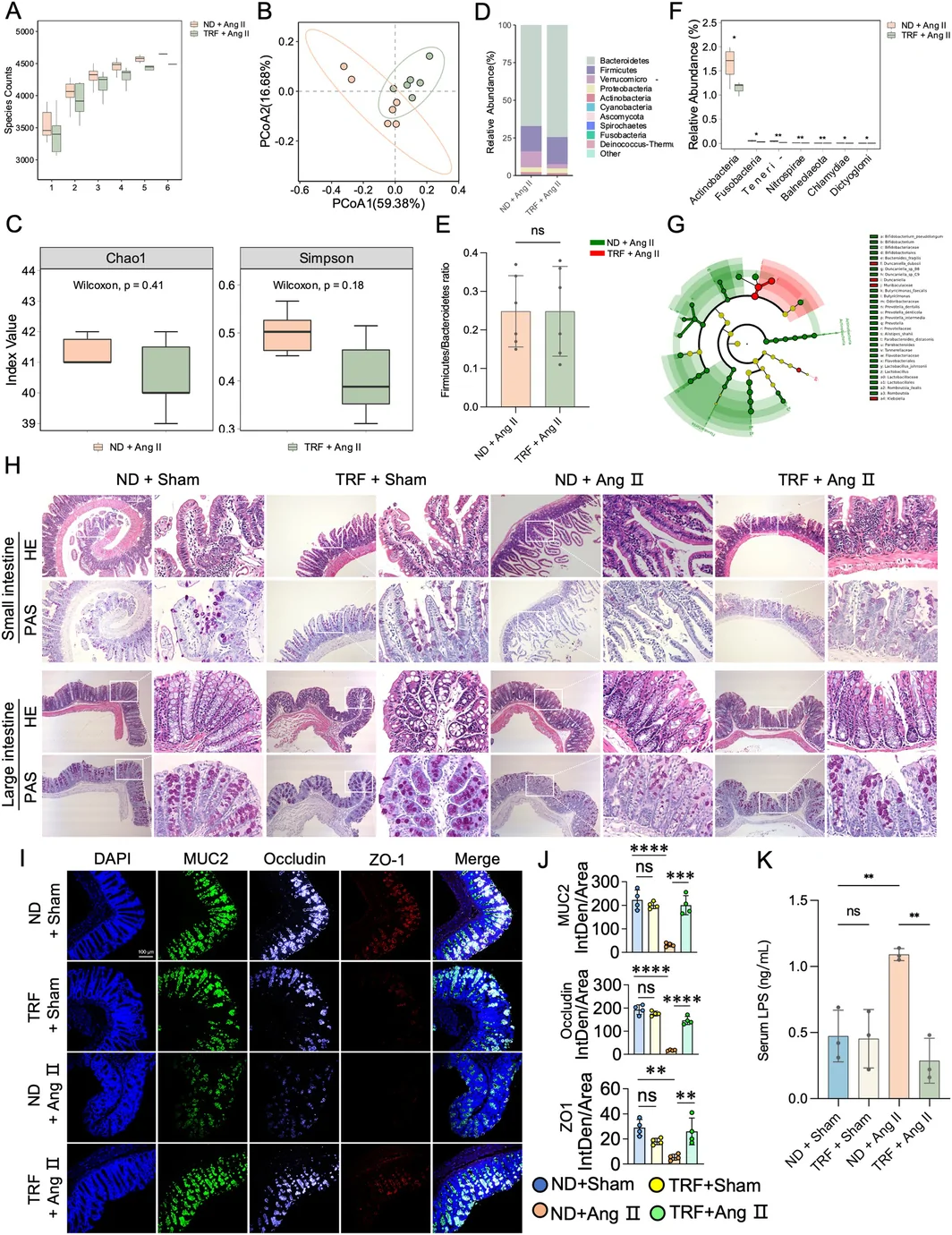
TRF reshaped a more stable gut microbiota and protected the intestinal barrier. (A) Species accumulation curve of fecal microbiota (after Ang II infusion). (B) PCoA scores based on the relative abundance of operational taxonomic units. Each symbol represents a sample (after Ang II infusion). (C) α‐diversity Chao1, Simpson, and Shannon indices (after Ang II infusion). (D) Dominant phyla in the ND or TRF group. (E) The column of F/B ratio (after Ang II infusion). (F) Differential analysis of phyla in the ND or TRF group (after Ang II infusion). (G) LEfSe analysis of two groups (after Ang II infusion). (H) HE and PAS staining images of large and small intestine tissues. (I) Representative immunofluorescence images of colon tissues stained with antibodies against Occludin, ZO‐1, and MUC2, scale bar =50 μm. (J) Fluorescence intensity analysis of (I). (K) Detection of LPS in blood, *n* = 3 per group. Sample numbers were *n* = 6 per group for (A–G) and *n* = 4 per group for (H) and (I). Results are expressed as mean ± SD, error bars represent SD. The scale bars of HE and PAS staining photographs were 200 μm (Low power field) or 50 μm (magnification field). *p < 0.05, **p < 0.01. ns, not significant.

### 3.3 TRF Attenuates Accelerated Vascular Aging‐Associated Pathological Cardiac Remodeling and Heart Failure

The interplay between accelerated vascular aging and cardiac dysfunction is insidious, with each process exacerbating the other. Heart failure, driven by pathological cardiac remodeling, is characterized by left ventricular (LV) hypertrophy, myocardial fibrosis, and inflammation (Nakamura and Sadoshima 2018). Following sustained Ang II infusion, mice exhibited cardiac morphological alterations, including increased heart size/weight, thickened ventricular walls, and reduced chamber dimensions. However, long‐term TRF intervention in the TRF + Ang II group significantly slowed the progression of pathological cardiac remodeling (Figure S1A,B,G). Noninvasive echocardiography was employed to assess cardiac structure, contractility, and hemodynamics. TRF + Ang II mice demonstrated superior cardiac function compared to accelerated vascular aging counterparts, as evidenced by improved ejection fraction (EF) and fractional shortening (FS) (Figure S1C–E). Additional echocardiographic parameters, such as interventricular septal thickness in systole (IVSs) and left ventricular internal diameter in systole (LVIDs), further corroborated TRF's protective effects against pathological remodeling (Table S2). Histopathological evaluation revealed that TRF intervention mitigated Ang II‐induced myocardial fibrosis, collagen deposition, and cardiomyocyte hypertrophy (Figure S1F,H–J). These findings collectively indicate that TRF not only alleviates accelerated vascular aging but also disrupts the vicious cycle of vascular‐cardiac crosstalk, thereby attenuating heart failure progression.

### 3.4 TRF Restructures a More Stable Gut Microbiota and Preserves Intestinal Barrier Integrity

Building on prior findings that TRF alters gut microbiota composition by increasing Firmicutes abundance (Figure 1F–H), we further investigated its effects on microbiota dynamics during Ang II‐induced accelerated vascular aging. Metagenomic sequencing of fresh fecal samples revealed distinct clustering between ND + Ang II and TRF + Ang II groups (Figure 3A,B). Ang II stimulation in ND mice markedly shifted microbial composition, reducing Bacteroidetes and elevating Verrucomicrobia. In contrast, TRF‐fed mice maintained stable Firmicutes/Bacteroidetes ratios before and after Ang II exposure (Figure 3D–F). While α‐diversity indices (Chao1 and Simpson) showed no intergroup differences (Figure 3C), LEfSe highlighted differential taxonomic dominance at phylum and genus levels (Figure 3G). Beyond nutrient absorption, the intestinal barrier critically prevents translocation of harmful microbes and toxins. Ang II‐induced accelerated vascular aging disrupted this barrier, exacerbating pathological progression. Histological analysis showed intact intestinal villi and normal goblet cell distribution in controls. Although villus architecture remained preserved in ND + Ang II mice, goblet cell numbers were significantly reduced, with diminished PAS‐stained glycogen content. TRF intervention restored goblet cell populations and glycogen reserves (Figure 3H). The paracellular barrier, regulated by tight junction proteins (occludin, ZO‐1, MUC2), was compromised in ND + Ang II mice. TRF prevented Ang II‐induced downregulation of these proteins, maintaining epithelial integrity (Figure 3I,J). LPS, a pro‐inflammatory endotoxin, enters systemic circulation upon barrier leakage. TRF + Ang II mice exhibited lower serum LPS levels, indicating preserved barrier function (Figure 3K). Notably, no differences in acute inflammatory markers C‐reactive protein (CRP) were observed across groups (Table S1), likely because LPS‐driven inflammation primarily targeted vascular tissues, aligning with the chronic, low‐grade inflammation characteristic of aging.

### 3.5 Microbiota Transplantation From TRF Mice Protects Against Ang II‐Induced Accelerated Vascular Aging

To establish causality between TRF‐modulated gut microbiota and vascular protection, we transplanted fecal microbiota from SPF mice fed ND or TRF for 10 weeks into GF mice, followed by Ang II‐induced accelerated vascular aging. No mortality occurred during microbiota transfer, and survival rates did not differ significantly among groups post‐Ang II induction (Figure S4B).

To verify the validity of the FMT model, we evaluated the gut microbiota structures of donor and recipient GF mice via PCoA based on Bray‐Curtis distances (Figure S15). ANOSIM analysis confirmed statistically significant structural differences among the groups (*R* = 0.594, *p* = 0.001). Despite the observed structural convergence in recipients, attributable to the “colonization bottleneck” and the shared Ang II stress, significant compositional differences persisted between the FMT‐ND and FMT‐TRF recipient groups. This confirmed successful engraftment of the functional signatures from the TRF donor microbiota, providing the microbiological foundation for the subsequent metabolic and cardiovascular protective effects.

Consistent with direct TRF intervention, transplantation of TRF microbiota caused no adverse effects on body weight, body mass index (Lee index), and fasting blood glucose (Figure S4C,D,G), or gastrointestinal symptoms (Figure S4E). Colon length, epididymal fat mass (Figure S4F,H), and baseline organ characteristics/biochemical markers remained comparable (Tables S3 and S4). Phenotyping revealed that GF mice receiving TRF microbiota (FMT‐TRF + Ang II) exhibited significantly attenuated systolic and diastolic blood pressure elevation compared to the FMT‐ND + Ang II group (Figure 4A–C). Aortic PWV, a hallmark of vascular stiffness, increased in FMT‐ND + Ang II mice versus sham controls but was markedly reduced by TRF microbiota transplantation (Figure 4D). The vascular weight‐to‐body weight ratio mirrored PWV trends, further confirming TRF microbiota's intrinsic capacity to counteract age‐related vascular stiffening (Figure 4E). Ultrasound imaging demonstrated aortic dilation in FMT‐ND + Ang II mice, which was mitigated in the FMT‐TRF + Sham group (Figure 4G,H). Histopathological staining (H&E, EVG, Masson) and TEM revealed luminal expansion, wall thickening, elastic fiber disruption, collagen deposition, and disorganized ECM in FMT‐ND + Ang II mice—all ameliorated by TRF microbiota transfer (Figure 4F,I). Endothelium‐dependent vasodilation, a critical indicator of vascular health, was impaired in FMT‐ND + Ang II mice, as shown by reduced mesenteric artery responsiveness to acetylcholine. TRF microbiota preserved endothelial function, underscoring its protective role against accelerated vascular aging (Figure 4J–L). At the molecular level, the western blot assay revealed that the feces of TRF‐transplanted mice could significantly reduce the increase in aging‐related indicators p53, p21, and p16 in the aorta induced by AngII (Figure S14C,D). These findings conclusively demonstrate that TRF‐reshaped gut microbiota alone suffices to attenuate Ang II‐induced hypertension, vascular stiffness, and pathological remodeling, independent of direct dietary intervention.

**FIGURE 4:**
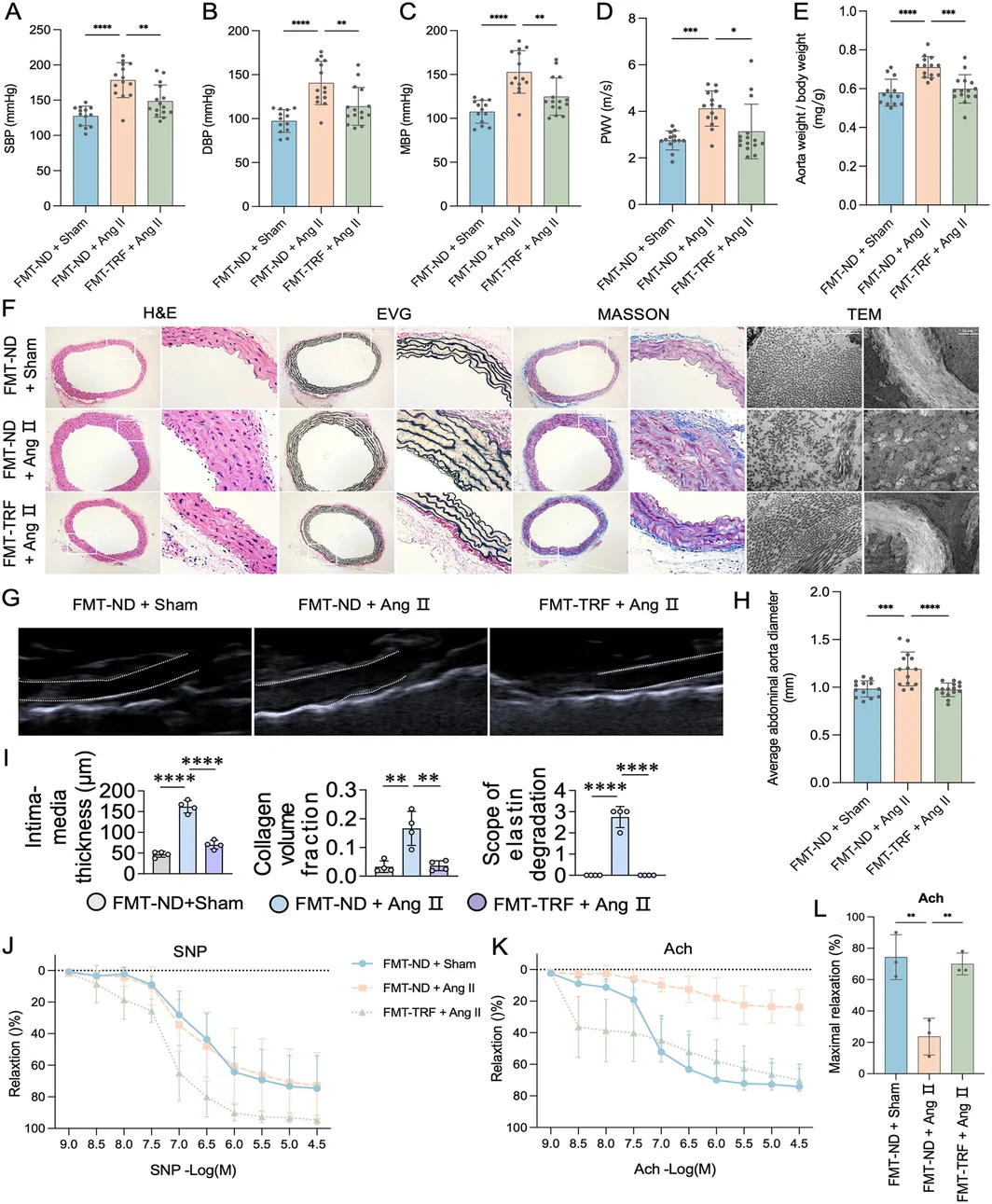
Vascular function after bacterial transplantation in germ‐free mice. (A) SBP of Ang II‐induced vascular aging germ‐free mice after FMT from mice fed with ND or TRF. (B) DBP of the three groups. (C) MBP of the three groups. (D) PWV of the three groups. (E) The aorta‐weight/body‐weight ratio of the three groups. (F) Representative images of the abdominal aorta stained with HE, EVG, Masson, and observed by TEM. TEM exhibits ultra‐microscopic collagen and elastic fiber arrangement in vascular wall, scale bar = 10 μm (Left, the cross section of collagen fibers; Right, the longitudinal section of elastic fibers). (G) Representative images of abdominal aorta ultrasound. (H) Diameter of abdominal aorta of the three groups. (I) Statistical analysis diagram of vascular wall thickness (HE), collagen deposition (MASSON) and elastic fiber damage (EVG). (J, K) Vascular ring assay to detect endothelium‐dependent (J) and endothelium‐independent (K) vasodilation. (L) Comparison of endothelium‐dependent and endothelium‐independent vasodilation responses to acetylcholine (Ach) and sodium nitroprusside (SNP), respectively. Relaxation was calculated as the percentage of the pre‐contraction tension induced by phenylephrine, with the stable pre‐contraction plateau set as 0% and full relaxation to baseline as 100%. Sample numbers of (A–E) and (H) were 13 (FMT‐ND + Sham), 14 (FMT‐ND + Ang II), and 15 (FMT‐TRF + Ang II). Sample numbers were *n* = 3 per group for (J–L) and *n* = 4 per group for (F) and (I). Results are expressed as mean ± SD, error bars represent SD. The scale bars of HE, EVG, MASSON staining photographs were 200 μm (Low power field) or 50 μm (magnification field). *p < 0.05, **p < 0.01, ***p < 0.001, ****p < 0.0001.

### 3.6 Microbiota Transplantation From TRF Mice Attenuates Vascular Aging‐Associated Pathological Cardiac Remodeling

Consistent with vascular findings, cardiac phenotyping of GF mice receiving FMT followed by Ang II induction revealed that TRF‐reshaped microbiota alleviated heart dysfunction. The FMT‐ND + Ang II group exhibited marked cardiac morphological alterations, including increased heart size/weight, ventricular wall thickening, and reduced chamber dimensions. In contrast, FMT‐TRF + Ang II mice showed slowed progression of pathological cardiac remodeling (Figure S2A,B,E). Histopathological analysis demonstrated that TRF microbiota transplantation mitigated Ang II‐induced myocardial fibrosis, collagen deposition, and cardiomyocyte hypertrophy (Figure S2F–I). Echocardiography further confirmed improved cardiac function in the FMT‐TRF + Ang II group, with enhanced EF and FS compared to vascular aging counterparts (Figures S2C,D and S4A). Additional parameters, such as interventricular septal thickness in systole IVSs and LVIDs, further supported the protective role of TRF microbiota against pathological cardiac remodeling (Table S5). These results underscore that TRF‐reshaped gut microbiota independently attenuates Ang II‐driven cardiac structural and functional deterioration, extending its therapeutic potential beyond direct dietary modulation.

### 3.7 TRF Microbiota Transplantation From TRF Mice Preserves Intestinal Barrier Integrity and Enhances SCFA Production in Ang II‐Induced GF Mice

Ang II induction in GF mice colonized with ND microbiota (FMT‐ND + Ang II) recapitulated intestinal barrier disruption and accelerated vascular aging. In contrast, FMT‐TRF + Ang II mice exhibited preserved intestinal architecture: intestinal villi structure and goblet cell distribution remained normal in FMT‐ND + Sham controls, whereas FMT‐ND + Ang II mice showed significant goblet cell depletion and reduced PAS‐stained glycogen content. TRF microbiota transplantation restored goblet cell numbers and glycogen reserves (Figure 5A). Tight junction proteins (Occludin, ZO‐1, MUC2), critical for paracellular barrier integrity, were downregulated in FMT‐ND + Ang II mice but maintained in the TRF microbiota group (Figure 5C,D). Correspondingly, serum LPS levels were lower in FMT‐TRF + Ang II mice (Figure 5B). TEM revealed intact epithelial tight junctions and a densely distributed glycocalyx on villus surfaces in FMT‐TRF + Ang II mice, mirroring sham controls and highlighting TRF microbiota's role in preserving barrier ultrastructure (Figure 5E). To explore mechanisms linking TRF microbiota to vascular protection, we analyzed SCFA—microbial metabolites pivotal for gut barrier and metabolic health. Targeted metabolomics showed distinct SCFA profiles across groups: FMT‐TRF + Ang II mice exhibited higher total SCFA levels and altered composition compared to FMT‐ND + Ang II counterparts (Figure 5F–H). Quantification of individual SCFA revealed significant increases in acetic acid, butanoic acid, valeric acid, and isobutyric acid in the TRF microbiota group (Figure 5I–L). These SCFA are known to enhance barrier integrity, regulate blood pressure, modulate glucose/lipid metabolism, and suppress inflammation—mechanisms likely central to TRF's anti‐aging effects (Hu et al. 2022; Mann et al. 2024).

**FIGURE 5:**
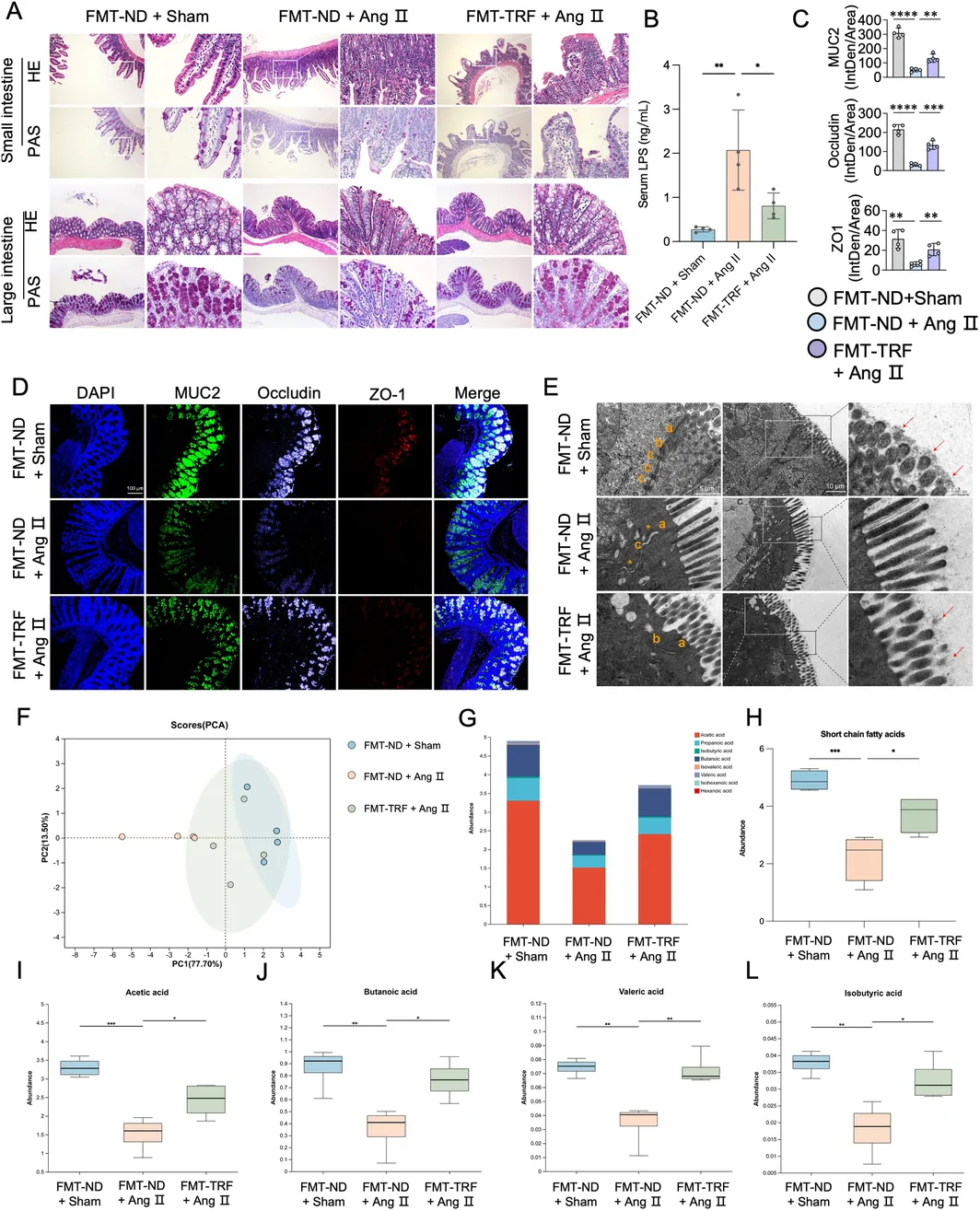
Delay in vascular aging was associated with an increase in SCFA production. (A) HE and PAS staining images of large and small intestine tissues (after FMT), scale bar = 200 μm. (B) Detection of lipopolysaccharide in blood (after FMT), *n* = 3 per group. (C) Fluorescence intensity analysis of (D). (D) Representative immunofluorescence images of colon tissues stained with antibodies against Occludin, ZO‐1, and MUC2 (after FMT), scale bar = 100 μm. (E) Observation of the intercellular connections of colon tissue by TEM (a, tight junction; b, adherens junction; c, desmosome. * Indicates a disrupted cell junction. The red arrow points to the glycocalyx). The scale bar was 5, 10, and 2.5 μm once from left to right. (F) Principal component analysis (PCA), scores based on the relative content. Each symbol represents a sample. (G) Composition of SCFA in feces of three groups of mice. (H) Comparison of total content of SCFA among the three groups. (I–L) Comparison of each SCFA among the three groups. Sample numbers were *n* = 7 per group for (F–L) and *n* = 4 per group for (A), (D), and (E). Results are expressed as mean ± SD, error bars represent SD. *p < 0.05, **p < 0.01, ***p < 0.001.

To further explore the association between FMT‐mediated protection and metabolic shifts, we analyzed the correlations between fecal acetic acid levels and key phenotypic endpoints in the FMT experiment. As shown in Figure S16A–I, fecal acetic acid concentrations were significantly positively correlated with colonic tight junction protein expression (ZO‐1, MUC2, Occludin) (*R* ^2^ > 0.46), and significantly negatively correlated with aortic senescence markers (p53, p16, p21), PWV, and cardiac hypertrophy/fibrosis parameters (*R* ^2^ > 0.35). These data provide additional quantitative evidence supporting acetic acid as a core metabolic effector of FMT‐induced protection.

### 3.8 Acetic Acid Is a Key Acid in TRF to Protect Intestinal Barrier and Delay Cardiovascular Aging by Remodeling the Gut Microbiota

To screen for the key SCFAs responsible for the protective effects of TRF, we performed integrated analyses of SCFA synthesis pathways based on metagenomic sequencing and targeted SCFA metabolomic profiles. The correlation heatmap revealed that TRF intervention ameliorated the Ang II‐induced reduction in the abundance of acetic acid synthesis pathway genes in both the TRF‐only and FMT models (Figure S5B). Individual SCFAs exhibited a strong association with their specific synthesis pathways, with acetic acid showing the most significant positive correlation with acetic acid‐generating pathways, such as the gene encoding acetic acid kinase (ackA) (Figure S5A). These analyses confirm that the elevation of acetic acid following TRF intervention is supported by specific metabolic pathways, establishing acetic acid as the core effector acid for subsequent functional studies.

To validate the protective effects of acetic acid on the intestinal barrier and cardiovascular system, mice were subjected to daily gavage of sodium acetate for 28 days in the Ang II‐induced vascular aging model. Acetate intervention significantly suppressed Ang II‐induced elevations in systolic, diastolic, and mean blood pressure (Figure 6A–C), reduced aortic stiffness (reflected by PWV) (Figure 6E), and attenuated the associated aortic dilation (Figure 6F,G), elastic fiber fragmentation (Figure 6I,K,M), collagen deposition (Figure 6H,L,M), and increased intima‐media thickness (Figure 6J,N). In the heart, Ang II induced prominent myocardial hypertrophy (Figure S8A,F,G,J), interstitial fibrosis (Figure S8F,H,I), and significant reductions in left ventricular ejection fraction (EF) and fractional shortening (FS) (Figure S8C–E). Acetate treatment effectively alleviated myocardial remodeling and improved cardiac systolic function. The ratios of aorta‐to‐body weight and heart‐to‐body weight were also reduced by acetate therapy (Figure 6D and Figure S8B). Furthermore, RT‐qPCR analysis confirmed that acetate treatment significantly reduced the mRNA levels of senescence‐related markers (p53, p21, and p16) in the aorta (Figure 6O). At the intestinal barrier level, multiple morphological assessments, including HE, PAS staining, and transmission electron microscopy, revealed that the number of goblet cells and the glycocalyx were significantly higher in the Acetate+Ang II group compared to the Ang II group, with notable improvements in microvillus structure and cellular junctions (Figure S5C,D). Acetate treatment also significantly upregulated the expression of colonic tight junction proteins (Occludin, ZO‐1, and MUC2) (Figure S5E). Collectively, these findings demonstrate that acetic acid confers robust protection against vascular aging, cardiac remodeling, and intestinal barrier dysfunction.

**FIGURE 6:**
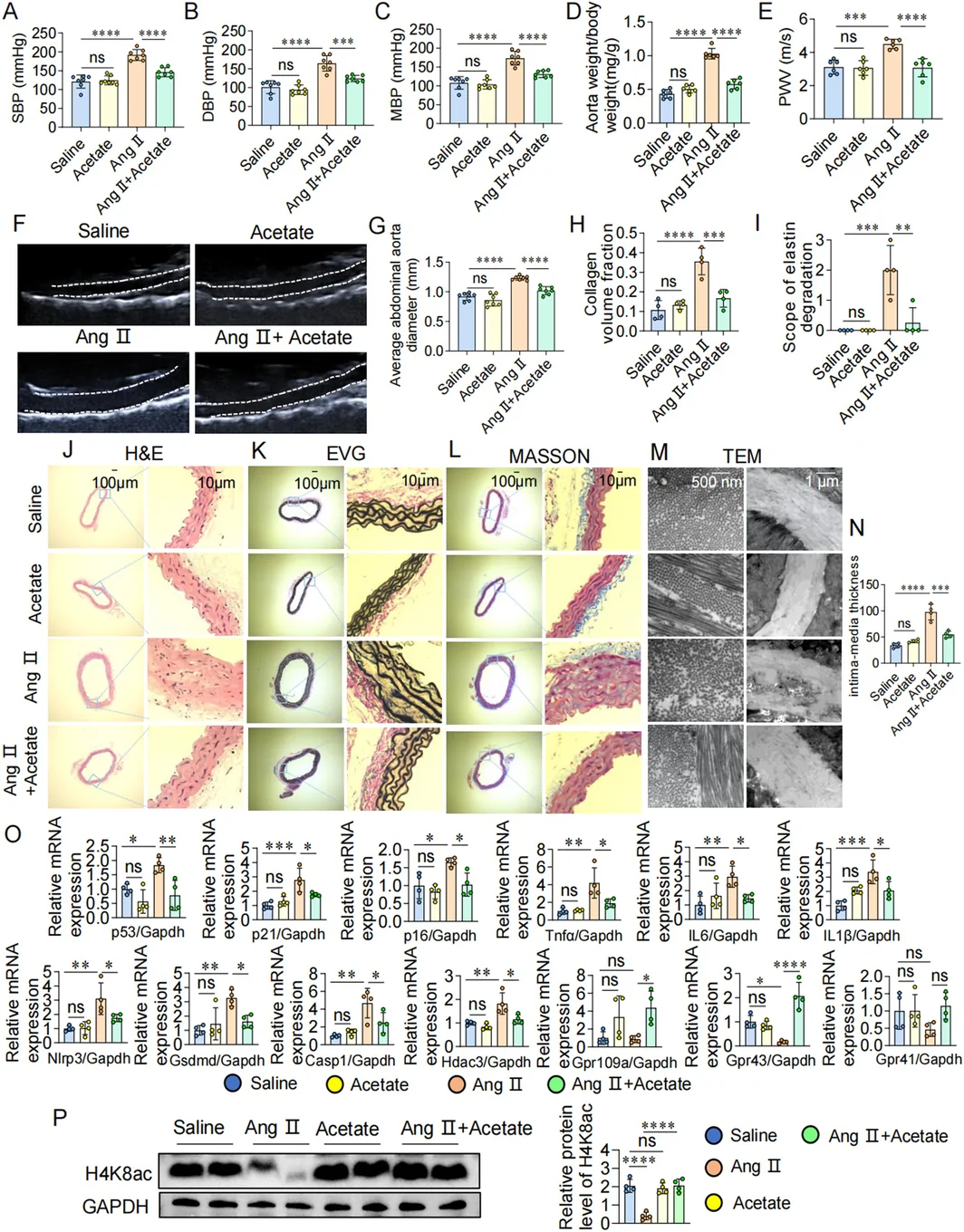
Sodium acetate intervention alleviates Ang II‐induced vascular aging and stiffening. (A–C) SBP, DBP, MBP in mice from each group. (D, E) Aorta weight‐to‐body weight ratio and aortic PWV. (F, G) Representative ultrasound images of the abdominal aorta and quantitative analysis of the average abdominal aortic diameter. (H, I) Statistical analysis diagram of collagen deposition (MASSON) and elastic fiber damage (EVG). (J–M) Representative HE, EVG, Masson staining (scale bars: 100, 10 μm), and TEM ultrastructural images of aortic cross‐sections (scale bars: 500 nm, and 1 μm). (N) Quantitative analysis of aortic IMT. (O) Relative mRNA expression levels of senescence‐related (p53, p21, p16), inflammatory (Nlrp3, Gsdmd, Casp1, Tnfα, IL6, IL1β, Hdac3), and SCFA receptor (Gpr109a, Gpr43, Gpr41) genes in aortic tissues. (P) Representative western blot images and quantitative analysis of H4K8ac and GAPDH in aortic tissues. (A–F) *n* = 6/group. (J–P) *n* = 4/group. *p < 0.05, **p < 0.01, ***p < 0.001; ns, not significant.

To determine whether the protective effects of TRF‐induced acetic acid are dependent on the gut microbiota, we disrupted the intestinal flora using an antibiotic cocktail (ABX) and subsequently performed sodium acetate supplementation via gavage. To evaluate the impact of this treatment on the microbiota, 16S rRNA sequencing was performed on fecal samples from the ABX validation experiment. Alpha diversity analysis revealed that TRF intervention did not significantly alter the Chao1 index but significantly increased the Simpson index, indicating that TRF primarily optimized community structure rather than increasing species number, consistent with the previous metagenomic results. Conversely, ABX treatment led to a sharp decline in the Chao1 index, confirming effective microbiota depletion (Figure S12A,B). Beta diversity analysis (PCoA) demonstrated highly significant structural differences among the four groups; the Ang II and TRF + Ang II groups were clearly separated along the PC1 axis, whereas the ABX and acetate‐supplemented groups highly overlapped in the ordination space, indicating that acetate supplementation failed to restore the microbial structure (Figure S12C). Phylum‐level analysis revealed that TRF significantly increased the Firmicutes/Bacteroidetes (F/B) ratio, which was disrupted by ABX treatment (Figure S12D). The Kruskal‐Wallis H test bar plot further elucidated the specific gut microbiota dysbiosis induced by ABX, characterized by significant depletion of commensal genera (e.g., *Blautia, Parabacteroides*) and abnormal enrichment of opportunistic pathogens (e.g., *Escherichia, Citrobacter*). Notably, although exogenous acetate supplementation failed to reverse this ABX‐induced structural imbalance (with the acetate group exhibiting a microbiota profile highly overlapping with the ABX group), it effectively improved cardiovascular phenotypes (Figure S12E). LEfSe analysis further revealed that the Ang II group harbored a pathological microbial fingerprint enriched in taxa such as *Bacteroidetes and Porphyromonadaceae*, which was successfully eliminated by TRF intervention (Figure S12F). Concurrently, targeted SCFA metabolomics on fecal samples from the ABX experiment revealed that antibiotic intervention, alongside depleting gut microbiota, significantly altered the fecal SCFA composition (Figure S12G). Inter‐group comparisons confirmed that acetic acid levels in the ABX‐treated group were significantly lower than those in the TRF + Ang II group, indicating that ABX effectively severed the microbiota‐dependent acetic acid supply, though acetic acid was not completely exhausted (Figure S12H).

Further experiments demonstrated that ABX‐induced microbiota depletion largely abrogated the protective effects of TRF against Ang II‐induced vascular remodeling and senescence (Figure S7A–L), cardiac dysfunction and remodeling (Figure S9A–J), and intestinal barrier injury (Figure S6A–C). Remarkably, exogenous sodium acetate supplementation in the ABX‐treated mice significantly reversed these pathological phenotypes, restoring blood pressure homeostasis, improving vascular and cardiac function, and reconstructing the gut barrier.

Additionally, in the acetate intervention model, the Ang II‐induced elevation of plasma LPS was effectively suppressed by acetate (Figure S5F); similarly, in the ABX rescue model, the abnormal LPS surge caused by ABX‐induced microbiota depletion was reversed by exogenous acetate supplementation (Figure S6D), further confirming that acetic acid blocks endotoxin translocation by repairing the intestinal barrier, thereby mitigating the sustained cardiovascular damage driven by systemic low‐grade inflammation. Finally, to ensure the safety of the interventions, we systematically monitored basal metabolism and multi‐organ morphology. No significant differences were observed in body weight, fasting blood glucose, epididymal adipose, colon length, gastrointestinal symptom or Lee index across groups (Figure S10G–L), and histopathological analyses of the liver, kidney, and spleen showed no obvious lesions (Figure S10A,B), confirming that the observed vascular and cardiac phenotypes were directly attributable to the cardiovascular effects of acetic acid rather than indirect metabolic influences.

### 3.9 *Limosilactobacillus* Is the Key Source Bacterium for Acetic Acid Elevation Following TRF Intervention

SCFAs produced by gut microbiota are critical regulators of host health, and identifying the specific acetic acid‐producing bacteria holds significant translational implications. To pinpoint the bacterial sources responsible for acetic acid elevation under TRF conditions, we conducted an integrated analysis combining 16S sequencing, targeted SCFA metabolomics, and previous metagenomic data from the ABX experiment. At the genus level, correlation heatmaps and scatter regression analyses revealed a significant positive correlation between the abundance of *Limosilactobacillus* and fecal acetic acid concentrations (Figure S13A,B). Although inter‐group comparisons in the 16S data did not reach statistical significance, TRF intervention increased the mean relative abundance of *Limosilactobacillus* in feces. Conversely, ABX antibiotic treatment not only eliminated the TRF‐induced enrichment of *Limosilactobacillus* but also led to a decrease in its mean abundance. Notably, in the exogenous acetate supplementation group (TRF + Ang II + ABX + Acetate), the abundance of *Limosilactobacillus* remained at extremely low levels, further corroborating that acetic acid production is highly dependent on the metabolic activity of *Limosilactobacillus* (Figure S13C). Correspondingly, metagenomic differential analysis revealed that TRF significantly increased the abundance of *Limosilactobacillus*, and the TRF + Ang II group exhibited an upward trend compared to the ND + Ang II group (Figure S13C). Furthermore, metagenomic analysis of FMT experiments showed that germ‐free mice receiving TRF donor microbiota also exhibited an upward trend in the mean abundance of *Limosilactobacillus* (Figure S13C). These data strongly suggest that *Limosilactobacillus* is a key bacterial source for intestinal acetic acid production following TRF intervention, acting as a vital upstream initiator in the TRF‐gut microbiota‐acetic acid‐intestinal barrier‐cardiovascular protection axis.

### 3.10 The GPR43/HDAC3/H4K8ac Signaling Axis and NLRP3 Inflammasome Constitute the Critical Molecular Mechanisms Underlying TRF‐Gut Microbiota‐ Acetic Acid Axis‐Mediated Cardiovascular Protection

The above results indicate that acetic acid may serve as a viable alternative to TRF. To further decipher the mechanisms of acetic acid, we first established an Ang II‐induced injury model in human umbilical vein endothelial cells (HUVECs) in vitro (Figure S11A). In vitro results demonstrated that Ang II stimulation significantly upregulated the mRNA expression of senescence‐associated genes (p53, p21, p16) and inflammatory cytokines (TNF‐α, IL‐6, IL‐1β, HDAC3), and activated the NLRP3/GSDMD/Casp1‐mediated pyroptosis pathway, accompanied by an overt senescent phenotype [increased senescence‐associated beta‐galactosidase (SA‐β‐gal) positive cells] (Figure S11B,C). Exogenous sodium acetate intervention effectively suppressed these gene expressions and significantly reduced the SA‐β‐gal positive rate. At the epigenetic level, Ang II induction led to a sharp reduction in the acetylation of histone H4 at lysine 8 (H4K8ac) in HUVECs, whereas acetate treatment effectively reversed this phenomenon and restored H4K8ac acetylation levels (Figure S11D). HDAC3 is a deacetylase whose expression and activity are typically inversely correlated with acetylation levels. Interestingly, sodium acetate treatment upregulated the expression of HDAC3 in HUVECs, a trend consistent with the changes in H4K8ac levels (Figure S11B). These in vitro data suggest that acetic acid may exert direct anti‐endothelial senescence and anti‐inflammatory effects through epigenetic regulatory pathways.

To further validate these mechanisms in vivo, we examined the expression of related proteins and mRNAs in aortic, cardiac, and colonic tissues (Figure 6O,P; Figures S5E, 6C, 7K,M, 8K,L, 9K,L and S14). In vivo western blot and PCR results confirmed that Ang II significantly upregulated the NLRP3/GSDMD/Casp1 pyroptosis pathway and inflammatory cytokines (TNF‐α, IL‐6, IL‐1β) in these tissues; conversely, TRF and acetate interventions effectively suppressed these alterations. At the receptor and epigenetic levels, the expression of the acetic acid‐specific receptor GPR43 in the aorta and heart was significantly increased in the TRF and acetate intervention groups, while histone deacetylase 3 (HDAC3) expression was substantially inhibited, consequently leading to a robust elevation in H4K8ac levels. These findings were consistent with the cellular‐level (Figure S11B). Notably, upon ABX‐induced gut microbiota depletion, the TRF‐induced upregulation of GPR43, downregulation of HDAC3, and elevation of H4K8ac in the aorta and heart were all reversed. However, exogenous sodium acetate supplementation fully restored GPR43 expression, re‐suppressed HDAC3, and reinstated H4K8ac levels. The activation and suppression of the NLRP3 inflammasome correspondingly changed inversely with these epigenetic markers. No clear association was observed in the expression trends of other SCFA receptors, such as GPR109A and GPR41, across the different animal models and cellular experiments. In summary, TRF produces acetic acid by remodeling the gut microbiota. Acetic acid may activate the cell‐surface GPR43 receptor, subsequently inhibit HDAC3‐mediated deacetylation of histone H4 at lysine 8, increase H4K8ac acetylation levels, and ultimately suppress NLRP3 inflammasome‐mediated pyroptosis and senescence, thereby contributing to cardiovascular protection.

## 4 Discussion

The renowned 17th‐century physician Thomas Sydenham once stated, “A man is as old as his arteries.” Modern medicine also unanimously agrees that cardiovascular and cerebrovascular diseases are the leading causes of serious long‐term disability and mortality among the elderly in developed countries (Lakatta and Levy 2003). For nearly a century, it has been known that caloric restriction can extend the lifespan of laboratory animals and ameliorate age‐related diseases. Recently, some alternative “anti‐aging” dietary patterns, such as the ketogenic diet, time‐restricted feeding, and protein restriction, have provided new mechanistic insights and potential clinical applications, but many questions remain regarding their efficacy (Lee et al. 2021). The present study elucidates a novel gut microbiota‐dependent mechanism by which TRF mitigates vascular aging, with SCFAs emerging as pivotal mediators linking microbial metabolism to vascular health. Our findings demonstrate that TRF not only restructures gut microbiota composition but also fortifies intestinal barrier integrity, thereby attenuating Ang II‐induced hypertension, vascular stiffness, and pathological cardiac remodeling. Critically, FMT experiments confirm that TRF‐reshaped microbiota alone suffices to recapitulate these protective effects, independent of direct dietary intervention. These results position the gut‐vascular axis as a central player in aging‐related cardiovascular dysfunction and highlight TRF as a non‐pharmacological strategy to counteract vascular senescence.

Central to TRF's benefits is its ability to enrich Firmicutes and stabilize the F/B ratio—a microbial signature associated with aging resilience (Mariat et al. 2009). A higher F/B ratio has been correlated with enhanced gut ecosystem stability and metabolic health in humans, potentially through increased SCFA production (Fernandes et al. 2014). Indeed, our metabolomic profiling revealed that TRF microbiota transplantation elevated fecal levels of acetic acid, butanoic acid, valeric acid, and isobutyric acid in Ang II‐treated mice. SCFAs, particularly butanoic acid, are renowned for their dual role in reinforcing intestinal barrier function via upregulation of tight junction proteins and suppressing systemic inflammation by inhibiting NF‐κB signaling (Liu et al. 2014; Lührs et al. 2002; Morrison and Preston 2016; Wang et al. 2012). In our study, acetic acid emerged as the key SCFA mediating these protective effects. The reduced serum LPS levels observed in TRF groups align with this mechanism, as SCFAs mitigate endotoxemia by preserving glycocalyx integrity and preventing bacterial translocation. These effects collectively dampen the low‐grade chronic inflammation that fuels vascular aging.

Notably, SCFAs extend their influence beyond the gut. As ligands for G protein‐coupled receptors (GPCRs) such as GPR41 and GPR43, SCFAs modulate blood pressure by regulating renin secretion and endothelial nitric oxide synthase (eNOS) activity (Li et al. 2021; Lymperopoulos et al. 2022; Natarajan et al. 2016). Butanoic acid, for instance, enhances endothelial function by inhibiting histone deacetylases, thereby promoting chromatin remodeling and anti‐oxidative gene expression (Chriett et al. 2019; Wang et al. 2024). It is a well‐known HDAC inhibitor, however, our data demonstrate that acetic acid, through GPR43/HDAC3/H4K8ac signaling, plays the predominant role in this model. This may explain the preserved endothelium‐dependent vasodilation and attenuated aortic stiffness in TRF mice, even under Ang II challenge. Furthermore, acetic acid's ability to suppress cardiomyocyte hypertrophy and collagen deposition—evidenced by improved cardiac EF/FS and reduced fibrosis—suggests systemic benefits that disrupt the vicious cycle of vascular‐cardiac crosstalk in aging.

Our FMT experiments provide causal evidence that TRF's vascular protection is microbiota‐dependent. GF mice colonized with TRF microbiota exhibited resistance to Ang II‐induced barrier disruption, hypertension, and ECM remodeling, mirroring outcomes seen in TRF‐fed mice. This underscores the therapeutic potential of targeting gut microbiota in aging populations, particularly those with limited capacity for dietary adherence. The elevated SCFA levels in TRF‐FMT mice further support the hypothesis that microbial metabolites serve as key executors of TRF's anti‐aging effects. However, while our study identifies SCFAs as critical mediators, the contribution of other microbial metabolites (e.g., bile acids, polyamines) warrants exploration.

While this study establishes a robust gut microbiota‐SCFA axis in TRF‐mediated vascular protection, several questions remain. First, human trials are needed to validate these findings, given interspecies differences in microbiota composition and SCFA metabolism. Second, although we identified *Limosilactobacillus* as the primary acetic acid‐producing bacterium enriched by TRF, strain‐level functional validation and mechanistic studies are needed to refine therapeutic microbiota engineering.

We also acknowledge several inherent limitations in the present study. First, our experiments exclusively utilized male mice. Given the well‐documented sexual dimorphism in both vascular aging and gut microbiota composition, it remains uncertain whether these findings are fully generalizable to females. Future studies incorporating female cohorts are essential to validate the applicability of our results across sexes. Second, regarding the assessment of systemic inflammation, while we measured tissue‐specific cytokines (IL‐6, TNF‐α, IL‐1β) via qPCR and analyzed serum LPS, relying solely on broad circulating markers like CRP might miss subtle, tissue‐level inflammatory variations. A more comprehensive cytokine panel would provide a clearer picture of the systemic anti‐inflammatory benefits. Third, although we established the GPR43/HDAC3/H4K8ac/NLRP3 axis in cardiovascular tissues, the exact downstream epigenetic targets of H4K8ac and the precise cross‐talk between HDAC3 and NLRP3 require further elucidation. Chromatin immunoprecipitation assays or similar approaches would be necessary to confirm whether H4K8ac directly modulates the NLRP3 promoter region or acts via secondary mediators. Fourth, while our FMT and ABX + Acetate experiments strongly implicate gut microbiota and acetic acid as the primary mediators, we cannot formally rule out synergistic contributions from other microbial metabolites, such as secondary bile acids and polyamines. Future metabolomics with a broader scope could help unravel these potential synergistic interactions.

Fifth, we acknowledge the lack of plasma SCFA quantification as a limitation of the present study. SCFAs are rapidly absorbed by the intestinal epithelium and subsequently cleared by the liver and peripheral tissues via first‐pass metabolism. Consequently, circulating SCFA levels in mice are typically very low and highly variable, making them a less reliable indicator of the intestinal microbiota's fermentation capacity compared to fecal SCFA levels. Therefore, in this study, we focused on fecal SCFA profiles to assess the production capacity of the gut microbiota. Nevertheless, future studies incorporating sensitive plasma SCFAs measurements would be valuable to further evaluate the systemic bioavailability and target organ delivery of these microbial metabolites.

Sixth, the clinical translation of our 6‐h TRF protocol warrants careful consideration. While a daily 6‐h feeding window is standard in murine models, this schedule must be adjusted when extrapolating to human populations. Rodents have a much higher metabolic rate and a shifted circadian phase relative to humans; consequently, a 6‐h feeding window in mice is generally considered roughly equivalent to an 8–10 h daily eating window in humans (such as early or late time‐restricted feeding). However, in elderly populations who often take multiple medications, have chronic comorbidities, or are at risk for malnutrition, implementing a strict 8–10 h window may still be challenging. Given these practical barriers, future studies should explore tailored TRF regimens—for example, adjusting feeding windows to accommodate medication schedules or adopting a less restrictive window (e.g., 10 h)—to better suit the needs of geriatric patients. Moreover, close monitoring for potential adverse effects, including hypoglycemia or sarcopenia, is essential before recommending TRF as a safe anti‐aging dietary intervention in frail elderly individuals.

Seventh, while the Ang II‐induced mouse model is well‐established for mimicking age‐related vascular dysfunction and effectively recapitulates key features of human vascular aging such as dilation, thickening, stiffness, and remodeling, its limitations must be acknowledged. As an acute, single‐factor‐induced model, it may not fully capture the polygenic and multifactorial nature of natural human aging. Consequently, we define it as an “accelerated vascular aging” model, acknowledging that the observed therapeutic effects might be overestimated compared to the complex, long‐term processes of natural aging. Future validation using naturally aged mice (e.g., 18–24 months old) would substantially strengthen the translational relevance of our findings. And our finding aligns with numerous pre‐clinical reports and some human observations, wherein TRF improves metabolic parameters and optimizes gut microbiota for cardiovascular benefit. However, there are also contradictory views. Some clinical studies report that intermittent fasting does not consistently outperform traditional caloric restriction in improving cardiometabolic outcomes. These discrepant efficacies may stem from multiple factors, including individual heterogeneity, specific dietary protocols, and the complexity of microbial metabolism.

## 5 Conclusions

In conclusion, TRF delays vascular aging by fostering a symbiotic gut microbiota that bolsters barrier function and amplifies SCFA production. These microbial metabolites act as multifunctional mediators, bridging gut health to cardiovascular resilience. Our work not only expands the mechanistic understanding of dietary interventions in aging but also positions microbiota‐targeted therapies as a promising avenue for combating age‐related vascular diseases.

## Author Contributions

Cuntai Zhang, Mian Cheng, and Tao Mi designed this study. Man Liu and Mandi Luo performed the study. Man Liu wrote the manuscript. Yangyang Huang, Tianyi Ji, Qian Wang, Jie Huang, Tao Jiang, and Yuman Wang contributed to the discussion. Man Liu analyzed the results. All authors approved the final version of the manuscript.

## Funding

This work was supported by the Noncommunicable Chronic Diseases‐National Science and Technology Major Project (grant no. 2024ZD0539100); the National Natural Science Foundation of China (grant no. 82371599 and 82070241); the Natural Science Foundation of Hubei Province (grant no. 2025AFB207); the National Natural Science Foundation Regional Innovation and Development Joint Fund (grant no. U24A20741); and the Zhang Cuntai Expert Workstation of Yunnan Province (grant no. 202405AF140057).

## Conflicts of Interest

The authors declare no conflicts of interest.

## Supporting information


**Figure S1:** Time‐restricted feeding (TRF) significantly reduced heart size and improved cardiac function.
**Figure S2:** Gut microbiota of TRF mice attenuated accelerated vascular aging‐associated cardiac pathological remodeling.
**Figure S3:** Evaluation of multiple physiological and metabolic parameters in mice after TRF and induced by Ang II.
**Figure S4:** Heart health, metabolic profile and gut health status of recipient mice following FMT.
**Figure S5:** Acetate intervention ameliorates Ang II‐induced intestinal barrier injury.
**Figure S6:** Antibiotic (ABX) intervention abolishes the intestinal barrier protective effects of TRF, which are restored by acetate supplementation.
**Figure S7:** Depletion of gut microbiota from ABX negates the vascular protective effects of TRF, whereas exogenous acetate reverses accelerated vascular aging phenotypes.
**Figure S8:** Acetate intervention attenuates Ang II‐induced pathological cardiac remodeling and dysfunction.
**Figure S9:** ABX intervention suppresses the cardiac protective effects of TRF, and exogenous acetate supplementation ameliorates pathological cardiac remodeling.
**Figure S10:** Safety assessment of acetate intervention and ABX treatment on basal metabolism and multi‐organ morphology in mice.
**Figure S11:** Acetate intervention protects human umbilical vein endothelial cells (HUVECs) against Ang II‐induced senescence, inflammation, and pyroptosis in vitro.
**Figure S12:** 16S rRNA sequencing and targeted SCFAs metabolomics analysis of fecal samples from four groups in the ABX experiment.
**Figure S13:** Integrated 16S ribosomal RNA sequencing, metagenomic and metabolomic analysis identifies the producing bacteria of acetic acid.
**Figure S14:** The TRF‐microbiota‐acetic acid axis confers cardiovascular protection via the GPR43/HDAC3/H4K8ac/NLRP3 signaling pathway.
**Figure S15:** PCoA validating the microbiota similarity between donor and recipient germ‐free mice.
**Figure S16:** Correlation analysis between fecal acetic acid concentrations and key outcome measures in the FMT experiment.
**Table S1:** Serological indicator in TRF experimental mice.
**Table S2:** Echocardiographic measurements in TRF experimental mice.
**Table S3:** General and metabolic characteristics of FMT mice.
**Table S4:** Serological indicator in the FMT mice.
**Table S5:** Echocardiographic measurements in FMT mice.
**Table S6:** Primers for reverse transcription quantitative polymerase chain reaction (RT‐qPCR).

## Data Availability

All the metagenomics sequencing data were submitted to the National Center for Biotechnology Information Sequence Read Archive (https://www.ncbi.nlm.nih.gov/) (accession number SRP579409). Additional data related to this paper may be requested from the authors. Email: manliu@hust.edu.cn (Man Liu).
