# Supplementary material for: Time‐Restricted Feeding Delays Cardiovascular Aging by Increasing Acetic Acid Through Remodeling Gut Microbiota
Source: Aging Cell. 2026 Oct 7;25(10):e70728. doi: 10.1111/acel.70728 (PMC13646369; doi:10.1111/acel.70728)
Supplement: Supplementary file 1 [file ACEL-25-e70728-s001.docx]

**Supporting Information for**

Time-restricted Feeding Delays Cardiovascular Aging by Increasing Acetic Acid through Remodeling Gut Microbiota

Man Liu^1,2†^, Mandi Luo^1,2†^, Yangyang Huang^1,2^, Tianyi Ji^1,2^, Yuman Wang^1,2^, Qian Wang^1,2^, Jie Huang^1,2^, Tao Jiang^1,2^, Cuntai Zhang^*1,2^ , Tao Mi^*1,2^, Mian Cheng^*1,2^

*Corresponding authors:

E-mail addresses: cheng.mian@icloud.com(Mian Cheng), mitao1023@163.com(Tao Mi), ctzhang0425@163.com/ ctzhang@tjh.tjmu.edu.cn (Cuntai Zhang).

^†^These authors have contributed equally to this work and share the first authorship

The word file includes:

Supporting Figure_1: Time-restricted feeding (TRF) significantly reduced heart size and improved cardiac function.

Supporting Figure_2: Gut microbiota of TRF mice attenuated accelerated vascular aging-associated cardiac pathological remodeling.

Supporting Figure_3: Evaluation of multiple physiological and metabolic parameters in mice after TRF and induced by Ang II.

Supporting Figure_4: Heart health, metabolic profile and gut health status of recipient mice following FMT.

Supporting Figure_5: Acetate intervention ameliorates Ang II-induced intestinal barrier injury.

Supporting Figure_6: Antibiotic (ABX) intervention abolishes the intestinal barrier protective effects of TRF, which are restored by acetate supplementation.

Supporting Figure_7: Depletion of gut microbiota from ABX treat negates the vascular protective effects of TRF, whereas exogenous acetate reverses accelerated vascular aging phenotypes.

Supporting Figure_8: Acetate intervention attenuates Ang II-induced pathological cardiac remodeling and dysfunction.

Supporting Figure_9：ABX intervention suppresses the cardiac protective effects of TRF, and exogenous acetate supplementation ameliorates pathological cardiac remodeling.

Supporting Figure_10：Safety assessment of acetate intervention and ABX treatment on basal metabolism and multi-organ morphology in mice.

Supporting Figure_11: Acetate intervention protects human umbilical vein endothelial cells (HUVECs) against Ang II-induced senescence, inflammation, and pyroptosis in vitro.

Supporting Figure_12: 16S rRNA sequencing and targeted SCFAs metabolomics analysis of fecal samples from four groups in the ABX experiment.

Supporting Figure_13: Integrated 16S ribosomal RNA sequencing‌, metagenomic and metabolomic analysis identifies the producing bacteria of acetic acid.

Supporting Figure_14: The TRF-microbiota-acetic acid axis confers cardiovascular protection via the GPR43/HDAC3/H4K8ac/NLRP3 signaling pathway.

Supporting Figure_15. PCoA validating the microbiota similarity between donor and recipient germ-free mice.

Supporting Figure_16. Correlation analysis between fecal acetic acid concentrations and key outcome measures in the FMT experiment.

Supporting Table_1: Serological indicator in TRF experimental mice.

Supporting Table_2: Echocardiographic measurements in TRF experimental mice.

Supporting Table_3: General and metabolic characteristics of FMT mice.

Supporting Table_4: Serological indicator in the FMT mice.

Supporting Table_5: Echocardiographic measurements in FMT mice.

Supporting Table_6: Primers for reverse transcription quantitative polymerase chain reaction (RT-qPCR)

**Supporting Figure 1. Time-restricted feeding (TRF) significantly reduced heart size and improved cardiac function**. (A) Heart morphology of the four groups of mice. The minimum scale in the ruler is 1 mm. (B) The heart-weight/body-weight ratio of angiotensin II (Ang II)-induced mice fed with normal diet (ND) or TRF. (C-D) The ejection fraction (EF) (%) and fractional shortening (FS) (%) of Ang II-induced mice fed with ND or TRF. (E) Typical images of Doppler ultrasound testing of cardiac function. (F) Quantitative analysis of cardiac collagen deposition and cardiomyocyte cross-sectional area. (G) Representative images of the heart stained with Hematoxylin-eosin (HE),Scale bar =1 mm (Low power field) or 50μm (magnification field). (H) Representative images of the heart stained with Masson, Scale bar =200 μm (Low power field) or 50μm (magnification field). (I) Representative images of the heart stained with Sirius Red, Scale bar =200 μm (Low power field) or 50μm (magnification field). (J) Representative images of the heart stained with Wheat germ agglutinin (WGA) (WGA, green; DAPI, blue), Scale bar =50 μm. Sample numbers were n = 10 per group for (A)-(E) and n = 4 per group for (F)-(J). Results are expressed as mean ± standard deviation (SD), error bars represent SD. ***p* < 0.01, *****p* < 0.0001. ns, not significant.

**Supporting Figure 2.** **Gut microbiota of TRF mice attenuated accelerated vascular aging-associated cardiac pathological remodeling**. After fecal microbiota transplantation (FMT), (A) heart morphology of the four groups of mice was examined. The minimum scale in the ruler is 1 mm. (B) The heart-weight/body-weight ratio of Ang II-induced mice fed with ND or TRF. (C) The EF (%) of Ang II-induced mice fed with ND or TRF. (D) The FS (%) of Ang II-induced mice fed with ND or TRF. (E) Representative images of the heart stained with HE, Scale bar =1 mm (Low power field) or 50μm (magnification field). (F) Representative images of the heart stained with Masson, Scale bar 200 μm (Low power field) or 50μm (magnification field). (G) Representative images of the heart stained with Sirius Red, Scale bar =200 μm (Low power field) or 50μm (magnification field). (H) Representative images of the heart stained with WGA (WGA, green; DAPI, blue), Scale bar = 200 μm. (I) Quantitative analysis of cardiac collagen deposition and cardiomyocyte cross-sectional area. Sample numbers of (A)-(D) were 13 (FMT-ND+Sham),14 (FMT-ND+Ang Ⅱ), and 15 (FMT-TRF+Ang Ⅱ). Sample numbers were n = 4 per group for (E)-(H). Results are expressed as mean ± SD, error bars represent SD. ****p* < 0.001, *****p* < 0.0001.

**Supporting Figure 3.** **Evaluation of multiple physiological and metabolic parameters in mice after** **TRF and induced by Ang II.** (A) Average food intake curve of the ND or TRF group, n = 20 per group. (B) Body weight of mice fed with ND or TRF after 10 weeks. (C) Lee’s index comparison among the ND and TRF group after 10 weeks. (D) Epididymal adipose weight of mice fed with ND or TRF after 10 weeks. (E) Fasting blood glucose of mice fed with ND or TRF after 10 weeks. (F) Curve of blood glucose levels during the oral glucose tolerance test (OGTT) in week 7. (G) Area under curve (AUC) of OGTT (mean ± SD). (H) Body weight of Ang II-induced mice fed with ND or TRF. (I) Lee index of Ang II-induced mice fed with ND or TRF. (J) Fasting blood glucose of Ang II-induced mice fed with ND or TRF. (K) Curve of blood glucose levels during the OGTT of Ang II-induced mice fed with ND or TRF, n = 15 per group. (L-N) Representative images of the liver (L), kidney(M), and spleen(N) stained with Hematoxylin-eosin (HE), Scale bars were 500 μm (Low power field) or 200μm (magnification field). (O) Pulsed-doppler sonograms of pulse wave velocity (PWV) detection of thoracic aorta (AO) and the abdominal aorta (AB) in mice, n = 15 per group. Sample numbers were n = 10 per group for (B)-(J) and n = 4 per group for (L)-(N). Results are expressed as mean ± SD, error bars represent SD. ns, not significant.

**Supporting Figure 4.** **Heart health, metabolic profile and gut health status of recipient mice following FMT.** (A) Typical images of Doppler ultrasound testing of cardiac function of Ang II-induced accelerated vascular aging germ-free mice after FMT from mice fed with ND or TRF. (B) Survival curve of mice. (C) Body weight changes of GF mice. (D) Fasting blood glucose changes of GF mice. (E) Anal features of mice reflecting diarrhea. (F) Representative images of the colon after 4 weeks of Ang II induction. (G) Lee’s index comparison among the three groups. (H) Representative images of epididymal fat. Sample numbers of (A)-(D), and (G) were 13 (FMT-ND+Sham),14 (FMT-ND+Ang Ⅱ), and 15 (FMT-TRF+Ang Ⅱ). Sample numbers were n = 4 per group for (E), (F), and (H). Results are expressed as mean ± SD, error bars represent SD. The minimum scale in the ruler is 1 mm. ns, not significant.

**Supporting Figure 5.** **Acetate intervention ameliorates Ang II-induced intestinal barrier injury.** (A) Correlation heatmap illustrating the association between short chian fatty acids (SCFAs) synthesis pathway abundance (metagenomics) and targeted SCFAs metabolomic profiles, showing a strong positive correlation between acetic acid and its synthesis pathways. (B) Box plots showing the abundance of acetic acid, butanoic acid, valeric acid, and isobutyric acid synthesis pathways across different treatment groups (metagenomics). (C) Representative HE and Periodic Acid-Schiff (PAS) staining images of the small intestine and large intestine (Scale bars: 100 μm and 20 μm). (D) Observation of the intercellular connections of colon tissue by transmission electron microscopy (TEM) (a, Tight junction; b, adherens junction; c, desmosome. * Indicates a disrupted cell junction. The red arrow points to the glycocalyx). The Scale bar was 500nm,1μm, and 500 nm once from left to right. (E) Plasma lipopolysaccharide (LPS) concentration. (F) Relative mRNA expression levels of barrier-related(Muc2, Occludin, Zo1), inflammatory (Nlrp3, Gsdmd, Casp1, Tnfα, IL6, IL1β, Hdac3) and SCFAs receptor (Gpr109a, Gpr43, Gpr41) genes in colon tissues determined by qPCR. Data are presented as mean ± SD. (A)-(B), n = 6/group. (C)-(F), n = 4/group **p* < 0.05, ***p* < 0.01, ****p* < 0.001 compared with the Saline, Ang II, or TRF+Ang II groups; ns, not significant.

**Supporting Figure 6. Antibiotic (ABX) intervention abolishes the intestinal barrier protective effects of TRF, which are restored by acetate supplementation.** (A) Representative HE and PAS staining images of the small intestine and large intestine in the TRF+Ang II, TRF+Ang II+ABX, and TRF+Ang II+ABX+Acetate groups (Scale bars: 100 μm and 20 μm). (B) Observation of the intercellular connections of colon tissue by TEM (a, Tight junction; b, adherens junction; c, desmosome. * Indicates a disrupted cell junction. The red arrow points to the glycocalyx). The Scale bar was 500nm,1μm, and 500 nm once from left to right. (C) Relative mRNA expression levels of barrier-related(Muc2, Occludin, Zo1), inflammatory (Nlrp3, Gsdmd, Casp1, Tnfα, IL6, IL1β, Hdac3) and SCFAs receptor (Gpr109a, Gpr43, Gpr41) genes in colon tissues determined by qPCR. (D) Plasma LPS concentration. Data are presented as mean ± SD. n=4/group. **p* < 0.05, ***p* < 0.01, ****p* < 0.001; ns, not significant.

**Supporting Figure 7. Depletion of gut microbiota from ABX treat negates the vascular protective effects of TRF, whereas exogenous acetate reverses accelerated vascular aging phenotypes.** (A) systolic blood pressure (SBP), diastolic blood pressure (DBP), and mean blood pressure (MBP) in the three groups (TRF+Ang II, TRF+Ang II+ABX, and TRF+Ang II+ABX+Acetate). (B) Aorta weight-to-body weight ratio. (C) PWV. (D) Representative ultrasound images of the abdominal aorta. (E–F) Average abdominal aortic diameter and aortic intima-media thickness (IMT). (G–J) Representative HE, Verhoeff's van Gieson (EVG), Masson staining (Scale bars: 100 μm, 10 μm), and TEM ultrastructural images of aortic cross-sections (Scale bars: 500 nm, and 1 μm). (K) Relative mRNA expression levels of senescence-related (p53, p21, p16), inflammatory (Nlrp3, Gsdmd, Casp1, Tnfα, IL6, IL1β, Hdac3), and SCFAs receptor (Gpr109a, Gpr43, Gpr41) genes in aortic tissues. (L) Statistical analysis diagram of collagen deposition (Masson) and elastic fiber damage (EVG). (M) Representative Western blot images and quantitative analysis of H4K8ac and GAPDH in aortic tissues. Data are presented as mean ± SD. (A)-(F), n = 6/group. (G)-(M), n = 4/group. **p* < 0.05, ***p* < 0.01, ****p* < 0.001; ns, not significant.

**Supporting Figure 8.** **Acetate intervention attenuates Ang II-induced pathological cardiac remodeling and dysfunction.** (A) Representative gross morphology of isolated hearts. (B) Heart weight-to-body weight ratio. (C–D) Left ventricular ejection fraction (EF) and fractional shortening (FS) determined by echocardiography. (E) Representative M-mode echocardiograms. (F) Quantitative analysis of cardiac collagen deposition and cardiomyocyte cross-sectional area. (G–I) Representative HE (Scale bars: 1 mm, 10 μm), Masson (Scale bars: 100 μm, and 20 μm), and Sirius red (Scale bars: 100 μm, and 20 μm) staining of cardiac tissue. (J) Representative DAPI/WGA fluorescence staining of cardiomyocyte membranes (Scale bar: 50 μm). (K) Relative mRNA expression levels of cardiac genes (Gpr109a, Gpr43, Gpr41, Nlrp3, Gsdmd, Casp1, Tnfα, IL6, IL1β, Hdac3). (L) Representative Western blot images and quantitative analysis of H4K8ac and GAPDH in cardiac tissues. Data are presented as mean ± SD. (A)-(E), n = 6/group. (F)-(L), n = 4/group. * *p* < 0.05, ***p* < 0.01, ****p* < 0.001; ns, not significant.

**Supporting Figure 9. ABX intervention suppresses the cardiac protective effects of TRF, and exogenous acetate supplementation ameliorates pathological cardiac remodeling.** (A) Representative gross morphology of isolated hearts from the TRF+Ang II, TRF+Ang II+ABX, and TRF+Ang II+ABX+Acetate groups. (B) Heart weight-to-body weight ratio. (C–D) EF and FS determined by echocardiography. (E) Representative M-mode echocardiograms. (F–H) Representative HE (Scale bars: 1 mm, 10 μm), Masson (Scale bars: 100 μm, and 20 μm), and Sirius red (Scale bars: 100 μm, and 20 μm), staining of cardiac tissue. (I) Representative DAPI/WGA fluorescence staining images. (J) Quantitative analysis of cardiac collagen deposition and cardiomyocyte cross-sectional area(Scale bar: 50 μm). (K) Representative Western blot images and quantitative analysis of H4K8ac and GAPDH in cardiac tissues. (L) Relative mRNA expression levels of cardiac genes (Gpr109a, Gpr43, Gpr41, Nlrp3, Gsdmd, Casp1, Tnfα, IL6, IL1β, Hdac3). Data are presented as mean ± SD. (A)-(E), n = 6/group. (F)-(L), n = 4/group. **p* < 0.05, ***p* < 0.01, ****p* < 0.001; ns, not significant.

**Supporting Figure 10. Safety assessment of acetate intervention and ABX treatment on basal metabolism and multi-organ morphology in mice.** (A–B) Representative HE staining images of the liver, kidney, and spleen from the acetate intervention experiment and ABX+acetate rescue experiment, respectively. (C–D) Epididymal fat weight. (E–F) Representative gross images of the colon and perianal region. (G–L) Dynamic changes in body weight, fasting blood glucose, and Lee index throughout the experimental period. No significant differences (ns) were observed between groups in basal metabolic parameters or organ morphology. n=6/group. Data are presented as mean ± SD.

**Supporting Figure 11. Acetate intervention protects human umbilical vein endothelial cells (HUVECs) against Ang II-induced senescence, inflammation, and pyroptosis in vitro.** (A) Schematic timeline of the HUVECs experimental protocol: treatment with Ang II (10 μmol/L) and sodium acetate (3 μmol/L) for 72 h. (B) Relative mRNA expression levels of senescence (p53, p21, p16), inflammatory (Tnfα, IL6, IL1β, Hdac3), pyroptosis-related genes (Nlrp3, Gsdmd, Casp1) and SCFAs receptor (Gpr109a, Gpr43, Gpr41) in HUVECs. (C) Representative images of senescence-associated β-galactosidase (SA-β-gal) staining and quantitative analysis of SA-β-gal-positive cells (Scale bar: 100 μm). (D) Representative immunofluorescence images of H4K8ac (Scale bar: 50 μm) and quantitative analysis of integrated optical density (IOD) per unit area. n=4/group. Data are presented as mean ± SD. **p* < 0.05, ***p* < 0.01, ****p* < 0.001; ns, not significant.

**Supporting Figure 12. 16S rRNA sequencing and targeted SCFAs metabolomics analysis of fecal samples from four groups in the ABX experiment.** (A) Alpha diversity analysis of gut microbiota. The Chao1 index (reflecting species richness) showed no significant change in the TRF intervention groups but sharply declined in the ABX-treated group. (B) Beta diversity analysis of gut microbiota. The Simpson index (reflecting species evenness) was significantly elevated in the TRF group, indicating that TRF optimized the microbial community structure. (C) Principal coordinate analysis (PCoA) of beta diversity based on Unweighted Unifrac distances. The four groups exhibited significant structural separation (R=0.744, p=0.001), with the TRF+Ang II and Ang II groups clearly separated along the PC1 axis, while the ABX group and the acetate supplementation group highly overlapped in the ordination space, indicating that acetate supplementation failed to restore the ABX-disrupted microbial structure. (D)Phylum-level composition analysis revealed that TRF significantly increased the Firmicutes/Bacteroidetes (F/B) ratio, which was disrupted by ABX treatment (Bacillota = Firmicutes; Bacteroidota = Bacteroidetes). (E) Kruskal-Wallis H test bar plot showing the mean proportion (%) of differentially abundant bacterial taxa (at genus and phylum levels) among the four groups (Ang II, TRF+Ang II, TRF+Ang II+ABX, and TRF+Ang II+ABX+Acetate). The P-values for inter-group comparisons are shown on the right side, revealing that the abundance distributions of multiple bacterial genera, including *Akkermansia, Porphyromonadaceae, Duncaniella, Bacteroidota, Bacteroides, Escherichia, Parabacteroides, Blautia, and Citrobacter*, exhibit statistically significant differences across the four groups. (F) LEfSe analysis identified a unique "pathological microbial fingerprint" enriched in the Ang II-induced group (e.g., *Bacteroidetes, Porphyromonadaceae*), which was successfully eliminated by TRF intervention. (G) Principal Component Analysis (PCA) scatter plot based on fecal SCFAs content. PC1 and PC2 explained 58.30% and 15.50% of the total variance, respectively. The results showed a clear spatial separation between the TRF+Ang II group and the Ang II group along the PC1 axis; however, the SCFAs metabolic profiles of the ABX-treated group (TRF+Ang II+ABX) and the exogenous acetate supplementation group (TRF+Ang II+ABX+Acetate) highly overlapped in two-dimensional space, indicating that exogenous acetate supplementation did not reverse the overall SCFAs compositional alterations induced by antibiotic-mediated microbiota depletion. (H) Inter-group comparison of fecal SCFAs concentrations, showing that acetic acid levels in the ABX-treated group were significantly lower than those in the TRF+Ang II group, confirming that ABX effectively severed the microbiota-dependent acetic acid supply. Data are presented as mean ± SD. **p* < 0.05, ***p* < 0.01, ****p* < 0.001; ns, not significant.

**Supporting Figure 13. Integrated 16S ribosomal RNA sequencing‌, metagenomic and metabolomic analysis identifies the producing bacteria of acetic acid.** (A) Correlation heatmap between the relative abundance of gut microbiota (16S ribosomal RNA sequencing‌) at the genus level and individual SCFA concentrations (SCFAs targeted sequencing‌) in TRF+Ang II and TRF+Ang II+ABX group mouse feces. Red and blue indicate positive and negative correlations, respectively. The red box highlights the significant positive correlation between *Limosilactobacillus* and acetic acid. (B) Scatter plot with linear regression analysis showing the correlation between *Limosilactobacillus* relative abundance (16S ribosomal RNA sequencing‌)and acetic acid concentration (SCFAs targeted sequencing‌ ) (R²=0.262, P=0.0105). (C-E) Inter-group comparisons of *Limosilactobacillus* abundance across different experimental models [C: 16S ribosomal RNA sequencing (Ang II, TRF+Ang II, TRF+Ang II+ABX , TRF+Ang II+ABX+Acetate); D: metagenomic sequencing (FMT models) ; E: metagenomic sequencing ( ND+Sham, TRF+Sham, ND+AngⅡ, TRF+ AngⅡ)]. n=6/group. **p* < 0.05, ****p* < 0.001; ns, not significant.

**Supporting Figure 14.** **The TRF-microbiota-acetic acid axis confers cardiovascular protection via the GPR43/HDAC3/H4K8ac/NLRP3 signaling pathway.** (A, E) Representative Western blot bands for NLRP3, GSDMD, CASP1, GPR109A, GPR43, GPR41, HDAC3, and H4K8ac in aortic and cardiac tissues (ND/TRF models). (B) Relative mRNA expression levels of aortic genes (ND/TRF models). (C, F) Representative Western blots of the aforementioned proteins in aortic and cardiac tissues from the FMT models. (D, G, H, I) Quantitative analysis of the relative protein expression levels of senescence markers (p53, p21, p16), pathway proteins (NLRP3, GSDMD, CASP1, GPR109A, GPR43, GPR41, HDAC3) and inflammatory factors (Tnfα, IL6, IL1β) in the aorta and heart (including ND/TRF and FMT models). n=4/group. Data are presented as mean ± SD. **p* < 0.05, ***p* < 0.01, ****p* < 0.001; ns, not significant.

**Supporting Figure 15. PCoA validating the microbiota similarity between donor and recipient germ-free mice.** In metagenomic sequencing, PCoA based on Bray-Curtis distances showing the fecal microbial community structures of ND/TRF donors and FMT recipients. analysis of similarities (ANOSIM) test revealed significant structural differences between groups (R = 0.594, *p* = 0.001). Although the recipient microbiota exhibited structural convergence to some extent, the statistical analysis confirmed significant compositional differences between the FMT-ND and FMT-TRF recipient groups, indicating successful engraftment of key signatures from the TRF donor microbiota.

**Supporting Figure 16.** **Correlation analysis between fecal acetic acid concentrations and key outcome measures in the FMT experiment.** Scatter plots showing the linear regression relationships between fecal acetic acid concentrations and indicators of intestinal barrier, vascular aging and cardiac remodeling in the FMT experiment. (A–C) Acetic acid levels were positively correlated with the integrated density (IntDen/Area) of colonic tight junction proteins (ZO-1, MUC2, Occludin). (D–G) Acetic acid levels were negatively correlated with aortic senescence-associated proteins (p53, p16, p21) and cardiomyocyte size. (H–I) Acetic acid levels were negatively correlated with aortic PWV and cardiac collagen volume fraction. Each data point represents an individual mouse (n = 4 per group, comprising FMT-ND+Sham, FMT-ND+AngⅡ, and FMT-TRF+AngⅡ groups). R² and P values were calculated using simple linear regression.

**Supporting Table 1. Serological indicator in TRF experimental mice** **(mean±SD).**

|  | ND + Sham  (n = 15) | TRF + Sham  (n = 15) | ND + Ang Ⅱ  (n = 15) | TRF + Ang Ⅱ  (n = 15) |
| --- | --- | --- | --- | --- |
| INS (pg/mL) | 127.60 ± 31.67 | 113.80 ± 35.94 | 91.81 ± 20.34^ns(a)^ | 119.90±30.77 ^ns(b)^ |
| GSP (mmol/L) | 3.87 ± 0.37 | 3.77 ± 0.53 | 3.95 ± 0.22 ^ns(a)^ | 3.81 ± 0.27 ^ns(b)^ |
| BUN (mg/dL) | 26.27 ± 6.35 | 26.51 ± 4.36 | 27.63 ± 2.78 ^ns(a)^ | 27.91 ± 4.81 ^ns(b)^ |
| CR (μmol/L) | 49.10 ± 6.77 | 62.50 ± 12.86 | 50.32 ± 3.09 ^ns(a)^ | 50.07 ± 3.12 ^ns(b)^ |
| AST (U/L) | 140.80± 22.22 | 164.40 ± 30.99 | 130.30 ± 26.21 ^ns(a)^ | 116.60±22.26 ^ns(b)^ |
| ALT (U/L) | 39.14 ± 5.20 | 40.30 ± 5.11 | 30.83 ± 5.56 ^ns(a)^ | 38.15 ± 8.18 ^ns(b)^ |
| ALB (g/L) | 29.07 ± 0.57 | 26.33 ± 4.27 | 27.75 ± 1.09 ^ns(a)^ | 26.28 ± 1.97 ^ns(b)^ |
| TC (mmol/L) | 2.38 ± 0.56 | 1.98 ± 0.49 | 2.33 ± 0.25 ^ns(a)^ | 4.25 ± 0.36 ^ns(b)^ |
| CRP (μg/mL) | 13.51 ± 2.31 | 10.91 ± 1.32 | 10.22 ± 4.71 ^ns(a)^ | 6.81 ± 0.94 ^ns(b)^ |

Abbreviations: ND, normal diet; Ang Ⅱ, angiotensin II; INS, insulin; GSP, glycated serum protein; BUN, blood urea nitrogen; CR, creatinine; AST, aspartate aminotransferase; ALT, alanine aminotransferase; ALB, albumin; TC, total cholesterol; CRP, C-reactive protein.

^ns^ No statistically significant difference

^(a)^ ND + Ang Ⅱ vs. ND + Sham

^(b)^ TRF + Ang Ⅱ vs. ND + Ang Ⅱ

**Supporting Table 2. Echocardiographic measurements in TRF experimental mice** **(mean±SD).**

|  | ND + Sham  (n = 10) | TRF + Sham  (n = 10) | ND + Ang Ⅱ  (n = 10) | TRF + Ang Ⅱ  (n = 10) |
| --- | --- | --- | --- | --- |
| IVSs (mm) | 1.21 ± 0.14 | 1.06 ± 0.09 | 0.95 ± 0.10***^(a)^ | 1.15 ± 0.19^#^^(b)^ |
| LVIDs (mm) | 2.25 ± 0.35 | 2.52 ± 0.46 | 3.22 ± 0.33****^(a)^ | 2.65 ± 0.29^##(b)^ |
| LVPWs (mm) | 1.08 ± 0.12 | 1.05 ± 0.11 | 0.94 ± 0.17 ^ns(a)^ | 1.06 ± 0.19^ns(b)^ |
| IVSd (mm) | 0.64 ± 0.16 | 0.58 ± 0.10 | 0.63 ± 0.10^ns(a)^ | 0.69 ± 0.13 ^ns(b)^ |
| LVIDd (mm) | 3.76 ± 0.34 | 3.91 ± 0.40 | 4.10 ± 0.37 ^ns(a)^ | 4.25 ± 0.36 ^ns(b)^ |
| LVPWd (mm) | 0.66 ± 0.14 | 0.67 ± 0.11 | 0.76 ± 0.15 ^ns(a)^ | 0.61 ± 0.18 ^ns(b)^ |

Abbreviations: ND, normal diet; Ang Ⅱ, angiotensin II; TRF, time-restricted feeding; IVSs, interventricular septal thickness in systole; LVIDs, left ventricular internal diameter in systole; LVPWs, left ventricular posterior wall thickness at end-systole; IVSd, interventricular septal thickness in diastole; LVIDd, left ventricular internal diameter at end-diastole; LVPWd, left ventricular posterior wall thickness at end-diastole.

*** *p* < 0.001

**** *p* < 0.0001

^#^ *p* < 0.00001

^##^ *p* < 0.000001

^ns^ No statistically significant difference

^(a)^ ND + Ang Ⅱ vs. ND + Sham

^(b)^ TRF + Ang Ⅱ vs. ND + Ang Ⅱ

**Supporting Table 3. General and metabolic characteristics of FMT mice (mean±SD).**

|  | FMT-ND+Sham  (n = 13) | FMT-ND+Ang Ⅱ  (n = 14) | FMT-TRF+Ang Ⅱ  (n = 15) | *P* value^a^ |
| --- | --- | --- | --- | --- |
| Body weight (g) | 29.40 ± 2.80 | 28.30 ± 2.30 | 29.50 ± 1.60 | 0.2638 |
| Liver weight (mg) | 1530.00 ± 310.20 | 1386.00 ± 288.80 | 1354.00 ± 207.40 | 0.2070 |
| Spleen weight (mg) | 87.10 ± 28.90 | 76.70 ± 17.30 | 75.60 ± 9.30 | 0.2556 |
| Heart weight (mg) | 135.20 ± 16.30 | 170.70 ± 33.60 | 139.70 ± 17.10 | 0.0005 |
| Epididymal adipose weight (mg) | 232.00 ± 49.90 | 184.50 ± 41.0 | 232.20 ± 86.40 | 0.0679 |
| Cecum weight (mg) | 184.90 ± 39.80 | 184.10 ± 29.0 | 190.20 ± 46.00 | 0.9037 |
| Colon length (cm) | 4.10 ± 0.50 | 14.60 ± 0.50 | 4.40 ± 0.60 | 0.0852 |

Abbreviations: ND, normal diet; Ang Ⅱ, angiotensin II; FMT: fecal microbiota transplantation.

^a^ FMT-TRF+Ang Ⅱ vs. FMT-ND+Ang Ⅱ

**Supporting Table 4. Serological indicator in the FMT mice (mean±SD).**

|  | FMT-ND + Sham  (n = 13) | FMT-ND + Ang Ⅱ  (n = 14) | FMT-TRF + Ang Ⅱ  (n = 15) |
| --- | --- | --- | --- |
| GSP (mmol/L) | 4.06 ± 0.40 | 3.93 ± 0.35 ^ns(a)^ | 3.85 ± 0.34^ns^^(b)^ |
| BUN (mg/dL) | 20.52 ± 4.09 | 26.46 ± 7.34 ^ns(a)^ | 22.65 ± 5.19 ^ns(b)^ |
| CR (μmol/L) | 38.25 ± 8.00 | 32.22 ± 2.86 ^ns(a)^ | 34.85 ± 6.22 ^ns(b)^ |
| AST (U/L) | 174.40 ± 42.54 | 225.90± 46.70^ns(a)^ | 166.00 ± 44.43^ns(b)^ |
| ALT (U/L) | 35.28 ± 5.20 | 66.22 ± 57.66 ^ns(a)^ | 37.41 ± 19.67 ^ns(b)^ |
| ALB (g/L) | 28.02 ± 2.67 | 29.87 ± 1.77 ^ns(a)^ | 26.14 ± 2.07 ^ns(b)^ |
| TC (mmol/L) | 2.57 ± 0.41 | 3.27 ± 0.50*^(a)^ | 2.38 ± 0.22^#(b)^ |

Abbreviations: ND, normal diet; Ang Ⅱ, angiotensin II; INS, insulin; TRF, time-restricted feeding; FMT, fecal microbiota transplantation; GSP, glycated serum protein; BUN, blood urea nitrogen; CR, creatinine; AST, aspartate aminotransferase; ALT, alanine aminotransferase; ALB, albumin; TC, total cholesterol.

^#^ *p* < 0.00001

* *p* < 0.05

^ns^ No statistically significant difference

^(a)^ FMT-ND + Ang Ⅱ vs. FMT-ND + Sham

^(b)^ FMT-TRF + Ang Ⅱ vs. FMT-ND + Ang Ⅱ

**Supporting Table 5. Echocardiographic measurements in FMT mice (mean±SD).**

|  | FMT-ND + Sham  (n = 13) | FMT-ND + Ang Ⅱ  (n = 14) | FMT-TRF + Ang Ⅱ  (n = 15) |
| --- | --- | --- | --- |
| IVSs (mm) | 1.35 ± 0.11 | 1.07 ± 0.14****^(a)^ | 1.23 ± 0.15^##(b)^ |
| LVIDs (mm) | 2.58 ± 0.33 | 3.20 ± 0.43****^(a)^ | 2.86 ± 0.17^#(b)^ |
| LVPWs (mm) | 1.18 ± 0.16 | 1.01 ± 0.13**^(a)^ | 1.10 ± 0.13^ns(b)^ |
| IVSd (mm) | 0.76 ± 0.32 | 0.70 ± 0.12 ^ns(a)^ | 0.71 ± 0.10 ^ns(b)^ |
| LVIDd (mm) | 4.40 ± 0.32 | 4.35 ± 0.49 ^ns(a)^ | 4.41 ± 0.24 ^ns(b)^ |
| LVPWd (mm) | 0.77 ± 0.12 | 0.78 ± 0.11 ^ns(a)^ | 0.75 ± 0.11 ^ns(b)^ |

Abbreviations: ND, normal diet; Ang Ⅱ, angiotensin II; FMT, fecal microbiota transplantation; IVSs, interventricular septal thickness in systole; LVIDs, left ventricular internal diameter in systole; LVPWs, left ventricular posterior wall thickness at end-systole; IVSd, interventricular septal thickness in diastole; LVIDd, left ventricular internal diameter at end-diastole; LVPWd, left ventricular posterior wall thickness at end- diastole.

** *p* < 0.01

**** *p* < 0.0001

^#^ *p* < 0.00001

^##^ *p* < 0.000001

^ns^ No statistically significant difference

^(a)^ FMT-ND + Ang Ⅱ vs. FMT-ND + Sham

^(b)^ FMT-TRF + Ang Ⅱ vs. FMT-ND + Ang Ⅱ

**Supporting Table 6.** **Primers for reverse transcription quantitative polymerase chain reaction (RT-qPCR).**

| Primer name | Species | Forward sequence | Reverse sequence |
| --- | --- | --- | --- |
| p53 | mouse | GCGTAAACGCTTCGAGATGTT | TTTTTATGGCGGGAAGTAGACTG |
| p21 | mouse | TCCACAGCGATATCCAGACA | GGACATCACCAGGATTGGAC |
| p16 | mouse | CGCAGGTTCTTGGTCACTGT | TGTTCACGAAAGCCAGAGCG |
| Nlrp3 | mouse | ATTACCCGCCCGAGAAAGG | TCGCAGCAAAGATCCACACAG |
| Gsdmd | mouse | TTCAGGCCCTACTGCCTTCT | GTTGACACATGAATAACGGGGTT |
| Casp1 | mouse | AATACAACCACTCGTACACGTC | AGCTCCAACCCTCGGAGAAA |
| Gpr109a | mouse | CTTGTTCAACTTGGCCGTGG | ATGAAGAGCATCACACGGCA |
| Gpr43 | mouse | TCCTTGATCCTCACGGCCTA | CGCCAGGGTCAGATTAAGCA |
| Gpr41 | mouse | CGGCTCACTGTAGTGTGGTT | AGTCGTACAGGCAGGAGGAT |
| IL1β | mouse | GCAACTGTTCCTGAACTCAACT | ATCTTTTGGGGTCCGTCAACT |
| IL6 | mouse | TAGTCCTTCCTACCCCAATTTCC | TTGGTCCTTAGCCACTCCTTC |
| Tnfα | mouse | AACTTCGGGGTGATCGGTCC | TGGGGGCTGGGTAGAGAATG |
| Hdac3 | mouse | ACCGTGGCGTATTTCTACGAC | CAGGCGATGAGGTTTCATTGG |
| Muc2 | mouse | AGGGCTCGGAACTCCAGAAA | CCAGGGAATCGGTAGACATCG |
| Occludin | mouse | TCTCTCAGCCAGCGTACTCT | TCCCATAGCCTCTGTCCCAA |
| Zo1 | mouse | TGAACGTCCCTGACCTTTCG | GCTCTGAACGTTGGTCAGGA |
| Gapdh | mouse | AGGTCGGTGTGAACGGATTTG | TGTAGACCATGTAGTTGAGGTCA |
| p53 | Human | CGCTTCGAGATGTTCCGAGA | CTTCAGGTGGCTGGAGTGAG |
| p21 | Human | GTCACTGTCTTGTACCCTTGTG | CGGCGTTTGGAGTGGTAGAAA |
| p16 | Human | CTTCGGCTGACTGGCTGG | TCATCATGACCTGGATCGGC |
| Nlrp3 | Human | GATCTTCGCTGCGATCAACAG | CGTGCATTATCTGAACCCCAC |
| Gsdmd | Human | GTGTGTCAACCTGTCTATCAAGG | CATGGCATCGTAGAAGTGGAAG |
| Caspase1 | Human | TTTCCGCAAGGTTCGATTTTCA | GGCATCTGCGCTCTACCATC |
| Gpr109a | Human | TGGGATACCTGGTGGCATTTAC | CTTCCGTCTTCGTGTCGTCT |
| Gpr43 | Human | GCCTTCAAGTCGTCACCTTCT | CATACATGGCGTTGATGCTG |
| Gpr41 | Human | GGTCGTGAGCGTGATCATAG | GGTCGTGAGCGTGATCATAG |
| IL1β | Human | ATGATGGCTTATTACAGTGGCAA | GTCGGAGATTCGTAGCTGGA |
| IL6 | Human | ACTCACCTCTTCAGAACGAATTG | CCATCTTTGGAAGGTTCAGGTTG |
| Tnfα | Human | CCTCTCTCTAATCAGCCCTCTG | GAGGACCTGGGAGTAGATGAG |
| Hdac3 | Human | CCTGGCATTGACCCATAGCC | CTCTTGGTGAAGCCTTGCATA |
| Gapdh | Human | ATTCCACCCATGGCAAATTCC | GACTCCACGACGTACTCAGC |

Abbreviations: Casp1, caspase 1; Gapdh, glyceraldehyde-3-phosphate dehydrogenase; Gpr41, G protein-coupled receptor 41; Gpr43, G protein-coupled receptor 43; Gpr109a, G protein-coupled receptor 109A; Gsdmd, gasdermin D; Hdac3, histone deacetylase 3; IL1β, interleukin 1 beta; IL6, interleukin 6; Muc2, mucin 2; Nlrp3, NLR family pyrin domain containing 3; p16, cyclin-dependent kinase inhibitor 2A; p21, cyclin-dependent kinase inhibitor 1A; p53, tumor protein p53; Tnf α, tumor necrosis factor-alpha; Zo1, zonula occludens-1

**Supporting Materials and methods**

**Acetic acid intervention and antibiotic cocktail (ABX) treatment animal model**

Animals and Experimental Grouping

Six- to eight-week-old specific pathogen-free (SPF) male mice were used in this study. After 1 week of acclimatization, mice were randomly divided into seven groups (n = 6 per group): (1) Saline; (2) Acetate; (3) Ang II; (4) Ang II+Acetate; (5) TRF + Ang II; (6) TRF + Ang II + ABX; and (7) TRF + Ang II + ABX + Acetate. The total modeling period spanned 35 days (from Day -7 to Day 28). All mice were housed in an SPF animal facility under controlled conditions (temperature: 20 ~ 26°C; humidity: 40%–70%; 12 h light/12 h dark cycle) with ad libitum access to common feed (The components are the same as those in the method section of the main text.) and water, except for the TRF groups. The detailed procedures for establishing the Ang II-induced accelerated vascular aging model and TRF protocol are described in the main text Materials and Methods and are not reiterated here. This TRF protocol was maintained from Day 1 to Day 28.

ABX treatment

To deplete the gut microbiota, mice were treated with a broad-spectrum ABX administered via the drinking water. The ABX solution consisted of ampicillin sodium (1 g/L), neomycin sulfate (1 g/L), metronidazole (1 g/L), and vancomycin hydrochloride (0.5 g/L) dissolved in sterile deionized water. All antibiotics were obtained from Shanghai Macklin Biochemical Co., Ltd.‌‌‌ To prevent light-induced degradation of the antibiotics, the ABX solution was prepared and stored protected from light, and freshly prepared ABX water was replaced every 48 hours to ensure sustained depletion of the gut microbiota. The ABX intervention was initiated during the acclimation period (Day -7) and maintained throughout the entire 35-day experimental period until the mice were euthanized. Control mice were administered an equivalent volume of sterile drinking water. At the end of the experiment, the efficiency of gut microbiota depletion was verified by 16S rRNA sequencing of fecal samples. Successful gut microbiota depletion was confirmed by 16S rRNA sequencing analysis of fecal samples (Supporting Figure 12 A and B).

Sodium acetate gavage

In the designated acetate-treated groups (Groups 2, 4, and 7), mice received a daily oral gavage of sodium acetate (AmBeed, USA) solution at approximately 9:00 AM from Day 1 to Day 28 (synchronized with the Ang II infusion period). The dosage of sodium acetate was 1.5g/kg/Day. Mice in the remaining groups received an equivalent volume of sterile water by gavage at the same time to serve as controls.

Sample Collection and Monitoring

During the experimental period, fasting blood glucose and body weight were measured weekly, following the protocols described in the main text Materials and Methods. At the conclusion of the experimental phases, the PWV, BP and cardiovascular ultrasound parameters were quantified in the mice. Approximately 50 mg of fresh fecal samples were collected from each mouse and divided into two cryogenic tubes (approximately 25 mg per tube). The samples were immediately snap-frozen in liquid nitrogen and subsequently stored at -80°C until further analysis. One tube was used for 16S rRNA sequencing to verify the efficiency of ABX-induced gut microbiota depletion, while the other was subjected to targeted LC-MS metabolomics to measure fecal SCFAs concentrations, with a particular focus on acetic acid levels. And then mice were euthanized and tissues, feces and blood samples were collected for subsequent experiments.

**Isolation and Culture of Primary HUVECs**

Primary HUVECs were isolated from fresh human umbilical cords obtained from healthy donors following delivery, with informed consent and approval by the Ethics Committee, adhering to the Declaration of Helsinki (Ethical Approval Number: TJ-IRB20230712). Under sterile conditions, the umbilical cord vein was flushed with phosphate-buffered saline (PBS) to remove residual blood. The vein lumen was then injected with 0.1% collagenase I (Sigma-Aldrich, USA) and digested in a 37 °C water bath for 15 min. The digestate was collected, and an equal volume of culture medium containing 10% fetal bovine serum (FBS, Gibco, USA) was added to terminate the digestion. After centrifugation at 1000 rpm for 5 min, the cell pellet was collected. The cells were resuspended in Endothelial Cell Medium (ECM, ScienCell, USA) supplemented with 5% FBS, Endothelial Cell Growth Supplement (ECGS), and 1% penicillin-streptomycin solution. Cells were seeded into gelatin-coated dishes and maintained in a 37 °C humidified incubator with 5% CO₂. The culture medium was changed every 2–3 days. Cells from passages 2 to 5 in the logarithmic growth phase were used for subsequent experiments.

**Ang II-induced senescence and acetate intervention in HUVECs**

HUVECs were seeded on day 1, and the medium was renewed on day 2. On Day 3, cells were treated with 10 µmol/L Ang II and/or 3 µmol/L sodium acetate for 72 hours (until day 6). Four groups were established: Saline, Ang II, Acetate, and Ang II + Acetate. After the treatment period, SA-β-gal staining, H4K8ac immunofluorescence, and RT-qPCR assays were performed.

**Total RNA Extraction from Tissues**

Mouse aorta and heart tissues were ground under liquid nitrogen or homogenized using a tissue homogenizer. Total RNA was extracted using TRIzol reagent (Invitrogen, USA) according to the manufacturer's instructions. Briefly, 1 mL of TRIzol was added to the tissue powder, mixed thoroughly, and incubated at room temperature for 5 min. Next, 200 μL of chloroform was added, shaken vigorously for 15 s, incubated for 3 min, and centrifuged at 12,000 g for 15 min at 4 °C. The upper aqueous phase was transferred to a new RNase-free tube, mixed with an equal volume of isopropanol, incubated for 10 min at room temperature, and centrifuged at 12,000 g for 10 min at 4 °C. The precipitate was washed twice with 75% ethanol (prepared with DEPC water). After air-drying, the RNA pellet was dissolved in RNase-free water. RNA concentration and purity (A260/A280 ratio between 1.8 and 2.0) were measured using a NanoDrop 2000 spectrophotometer (Thermo Scientific, USA) and stored at -80 °C.

**Total RNA Extraction from Cells**

After treatment, HUVECs were washed twice with pre-chilled PBS. TRIzol reagent (1 mL per 10 cm² culture area) was added to the culture dish, and cells were lysed by repeated pipetting. Subsequent steps, including chloroform extraction, isopropanol precipitation, ethanol washing, and RNA dissolution, were identical to the tissue RNA extraction protocol. The extracted RNA was used for reverse transcription reactions.

**Quantitative Real-Time PCR (qPCR)**

First-strand cDNA was synthesized from 1 μg of total RNA using the PrimeScript™ RT Reagent Kit (Takara, JAPAN) according to the manufacturer's protocol. qPCR was performed using TB Green® Premix Ex Taq™ II (Takara, JAPAN) on a real-time PCR detection system (Applied Biosystems StepOnePlus)(ThermoFisher, USA). The 20 μL reaction mixture consisted of 10 μL of 2× SYBR Green Mix, 0.5 μL of each forward and reverse primer, 2 μL of cDNA template, and RNase-free water. The thermal cycling protocol was as follows: pre-denaturation at 95 °C for 30 s; followed by 40 cycles of 95 °C for 5 s and 60 °C for 34 s. A melting curve analysis was performed at the end of the cycles to ensure amplification specificity. GAPDH was used as the internal reference gene to normalize for variations in RNA input and reverse transcription efficiency. The relative mRNA expression level of each sample was first calculated as the 2-ΔCt (Where ΔCt = Ct _target_-Ct _GAPDH_). To analyze the distribution of expression across different groups, these values were further normalized by dividing each value by the sum of all group values, resulting in a relative proportion where the sum of all groups equals 1 (or 100%). Primers used are listed in **Supporting Table 6**.

**SA-β-gal Staining**

SA-β-gal staining of HUVECs was performed according to the instructions of the SA-β-gal staining kit (Beyotime, China). Briefly, the culture medium was removed, and cells were washed once with PBS. The cells were fixed with 1 mL of fixative solution for 15 min at room temperature. After washing three times with PBS (3 min each), a staining working solution containing X-gal, staining buffer, and β-galactosidase staining solution was added to each well. Cells were incubated in a CO₂-free incubator at 37 °C in the dark overnight (16 h). After removing the staining solution and washing twice with PBS, cells were observed and photographed under an inverted microscope. At least 5 random fields were selected to count the percentage of dark blue-stained (SA-β-gal-positive) cells. Quantitative analysis was performed using ImageJ software.

**Cell immunofluorescence Staining**

HUVECs were seeded in confocal dishes. After treatment, cells were washed twice with PBS, fixed with 4% paraformaldehyde (PFA) for 20 min at room temperature, and permeabilized with 0.2% Triton X-100 for 10 min. After washing with PBS, cells were blocked with 5% bovine serum albumin (BSA) for 1 h at room temperature. Cells were then incubated with primary anti-H4K8ac antibody (Abclonal, A28114, China) (1:100) in a humidified chamber overnight at 4 °C. After washing, the cells were incubated with a fluorescence-conjugated secondary antibody Cy3-conjugated Goat anti-Rabbit IgG(H+L) (Abclonal, AS007, China), diluted (1:200) for 1 h at 37 °C in the dark. Finally, nuclei were counterstained with DAPI (Beyotime, China) for 5 min. Images were acquired using a confocal laser scanning microscope (Nikon, JAPAN). The integrated optical density (IOD) per unit area was quantified using ImageJ software.

**Full-length 16S rRNA gene sequencing and bioinformatics analysis**

Full-length 16S rRNA gene sequencing of mouse fecal samples was performed by Majorbio Bio-Pharm Technology Co., Ltd. (Shanghai, China) using the PacBio third-generation sequencing platform. Fresh fecal samples from mice were collected and immediately snap-frozen in liquid nitrogen, then sent to Majorbio Bio-Pharm Technology Co., Ltd. (Shanghai, China) for full-length 16S rRNA gene sequencing. Total genomic DNA was extracted from fecal samples using the E.Z.N.A.® Soil DNA Kit (Omega Bio-tek, USA) according to the manufacturer's protocols. DNA concentration and purity were assessed using a NanoDrop 2000 spectrophotometer (Thermo Scientific, USA), and DNA integrity was evaluated by 1% agarose gel electrophoresis. The full-length 16S rRNA gene was amplified using the universal primers 27F (5'-AGRGTTTGATYNTGGCTCAG-3') and 1492R (5'-TACGGYTACCTTGTTACGACTT-3'). After purification of the amplicons, sequencing libraries were constructed following the standard PacBio SMRTbell library preparation protocol. Qualified libraries were sequenced on the PacBio Sequel platform using single-molecule real-time (SMRT) sequencing technology.

Raw sequencing data were initially demultiplexed according to the barcode sequences, followed by length filtering and orientation correction, with sequences ranging from 1000 to 1800 bp retained for subsequent analyses. Denoising of the quality-controlled and assembled sequences was performed using the DADA2 plugin within the QIIME2 pipeline to generate amplicon sequence variants (ASVs). Taxonomic assignment of ASVs was carried out using the classify-consensus-blast method in QIIME2 against the NT_Taxon_core_v2024/16s_bacteria gene database. Sequences annotated as chloroplasts or mitochondria were removed to eliminate potential contamination. To minimize the impact of sequencing depth variation, the abundance data from 24 samples were rarefied to an even depth of 8,260 sequences per sample, yielding a total of 698 ASVs for subsequent analysis.

Alpha diversity indices (including Shannon, Simpson, Chao1, and ACE) and Beta diversity analyses were calculated using Mothur software (version 1.30.2), with visualizations performed using R software (version 3.3.1). PCoA based on Bray-Curtis distances, combined with ANOSIM and Adonis tests, was utilized to evaluate overall structural differences in the bacterial community among groups. Venn diagrams (version 2.4.3) were generated to identify unique and shared species across groups, and community bar plots and heatmaps were constructed to visualize species distribution patterns. For differential species screening, the Kruskal-Wallis test (for multiple groups) or Wilcoxon rank-sum test (for two groups) was performed using R software, and LEfSe (Linear Discriminant Analysis Effect Size) analysis was further applied to identify taxa with the greatest contribution to inter-group differences. The Random Forest machine learning algorithm was employed to identify potential biomarkers capable of significantly distinguishing different groups.

To explore the associations between the microbial community and physiochemical factors, redundancy analysis (RDA) or canonical correspondence analysis (CCA) was conducted. The Vegan package in R was used to perform variation partitioning analysis (VPA) to elucidate the major clinical or physiochemical factors constraining the microbial community. Functional prediction of the 16S rRNA data was performed using PICRUSt2 software (version 2.2.0). All bioinformatics analyses were performed on the Majorbio Cloud Platform (https://v.majorbio.com).
